# Clustering of multimorbidity in stroke and transient ischaemic attack survivors: a population-based study

**DOI:** 10.1186/s12916-026-04924-7

**Published:** 2026-05-18

**Authors:** Efthalia Massou, Duncan Edwards, Yajing Zhu, Zhirong Yang, Jonathan Mant

**Affiliations:** 1 The Primary Care Unit, Department of Public Health and Primary Care, University of Cambridge, Cambridge, UK; 2 Novo Nordisk R&D Digital Hub, 1 Pancras Square, Pancras Rd, London, N1C 4AG UK; 3 Department of Computational Biology and Medical Big Data, Faculty of Computer Science and Artificial Intelligence, Shenzhen University of Advanced Technology, Shenzhen, 518107 China; 4 Center for AI in Medicine, Artificial Intelligence Research Institute, Shenzhen University of Advanced Technology, Shenzhen, 518107 China

**Keywords:** Stroke, Multimorbidity, Long Term Conditions, Latent Class Analysis, Clustering, Age Stratification

## Abstract

**Background:**

Stroke and transient ischaemic attack (TIA) survivors frequently experience multiple long-term conditions (multimorbidity) placing substantial demands on healthcare systems. Moving away from a single-disease approach could lead to more efficient and effective care for stroke survivors with multimorbidity. However, it is unclear how to categorise the stroke population to achieve this. To inform care, this population-based study identified and described clusters of stroke survivors with multimorbidity.

**Methods:**

Using the Clinical Practice Research Datalink GOLD database, we identified 69,372 adult stroke/TIA survivors who were currently registered with a general practice on the 1st July 2017. We defined multimorbidity as the co-occurrence of ≥ 2 of 36 long-term conditions (including stroke/TIA) and divided patients into four age strata (< 45, 45–64, 65–84, ≥ 85). Within each stratum, Latent Class Analysis identified classes of co-morbid stroke survivors based on statistical diagnostics, clustering interpretation and clinical input. We investigated the validity of our findings, using a training and a test set. We described clusters according to prevalence of long-term conditions, demographic factors (age, gender, ethnicity, smoking, BMI) and health outcomes (mortality, hospital admissions, primary care consultations, prescriptions).

**Results:**

In the UK, 94·7% of adult stroke/TIA survivors live with at least one additional long-term condition, with a median of five conditions per patient. We identified 12 clusters. Among 45–64-year-olds, the “alcohol and substance misuse” (6%) and the “established cardiovascular disease” (5%) clusters, have the highest mortality, while the “lower morbidity, better outcomes” cluster included 49% of patients. In ages 65–84, the “poor mental health” cluster (25%) exhibits the highest mortality. Among patients $\:\ge\:$85, the “dementia-dominant” cluster had the highest rates of mortality, whereas the “established cardiovascular disease” cluster had the most hospital admissions. In each age strata, a cluster with increased mental health needs, and another with lower rates of multimorbidity and the best health outcomes could be identified.

**Conclusions:**

Internally validated clusters of stroke survivors can be identified with distinct patterns of multimorbidity, healthcare utilisation, and mortality. By understanding these clusters, more targeted, efficient, and integrated models of post-stroke care can be designed to better address the overall healthcare needs of stroke survivors.

**Supplementary Information:**

The online version contains supplementary material available at https://doi.org/10.1186/s12916-026-04924-7.

## Background

With more than 12 million new cases and 101 million prevalent cases, stroke is among the major causes of long-term disability worldwide. Its burden is rising due to ageing populations and improved post-stroke survival [1]. In the United Kingdom, 113,000 people are added each year to the around 1 million stroke survivors of the country [2]. Stroke and transient ischaemic attack (TIA) survivors often experience additional chronic conditions, with prevalence ranging from 43% to 94%, depending on definitions used [3].

Multimorbidity - the co-occurrence of two or more long-term conditions (LTCs) [4] is a major challenge for healthcare services. In the UK, it is estimated that 25% of adults experience multiple LTCs [5], with similar prevalence reported in other developed countries [6]; and prevalence is increasing [7]. This rising burden is partially attributed to increased life expectancy over the last decades [8, 9], driven by advances in prevention, curative medicine, and healthcare, particularly benefiting stroke/ TIA patients through improved treatment and rehabilitation [10].

For patients with multimorbidity, managing multiple chronic conditions often requires frequent healthcare visits and comprehensive care services, including treatment, social support, and polypharmacy management [11]. While the number, nature and severity of the LTCs influence service needs, studies consistently link multimorbidity to greater healthcare use [12, 13]. A previous study estimated that 27% of the adult UK population has multimorbidity and these patients account for 53% of general practitioner (GP) consultations, 79% of prescriptions, and 56% of hospital admissions [5].

While many organizations [14, 15] and researchers [16–18] have emphasized the importance of investigating multimorbidity patterns, no research has examined how multimorbidity is structured among stroke and TIA survivors. This limits understanding of their healthcare needs and therefore what would be optimal service. This study aims to fill this gap by identifying common clusters of multimorbidity within stroke/ TIA survivors.

## Methods

### Data source and study design

To investigate multimorbidity within a representative sample of the UK stroke/TIA population, this study used data from the Clinical Practice Research Datalink (CPRD) GOLD, a clinical primary care database representative of the UK population by age, sex and ethnicity [19].

We linked the data with patient- and practice-level area socioeconomic deprivation data (Index of Multiple Deprivation, IMD), and patient-level data on hospital admissions (Admitted Patient Care Hospital Episodes Statistics) [20].

The study protocol (20_056R) received scientific and ethical approval from CPRD Independent Scientific Advisory Committee.

### Study population

We included 69,372 adults (≥ 18 years) who were alive with a history of a stroke/TIA on the 1st July 2017 and were registered with a GP practice contributing at least one year up-to-standard data to CPRD by that date. Mortality and health service utilisation were assessed over a 2-year follow up period. This follow up period was chosen to provide sufficient time to capture mortality and health services utilisation outcomes while minimising the risk due to patients transferring practices, data quality variations over time, or changes in clinical practices (including the coronavirus pandemic from 2020). Stroke cases were identified using Read Codes recorded in primary care (Additional file 1: Table S1) [21].

### Definitions of patient’s characteristics, morbidities, and outcomes

Multimorbidity was operationalised using a validated list of 36 chronic conditions (Additional file 1: Table S2) widely used in UK primary care [5, 22–46]. These chronic conditions have significant impact on patient’s quality of life, use of services, morbidity and mortality and their use ensures methodological consistency and comparability with prior research.

Patient characteristics included gender, age, body mass index (BMI), smoking status, ethnicity, and socioeconomic status, as these are key demographic factors associated with multimorbidity and stroke prevalence [5, 41, 47]. Smoking status was categorised into three groups: current, ex-, and never smokers. Invalid BMI records (i.e., weight< 30 kg or ≥300 kg; height < 1.0 m or > 2.5 m) were excluded. BMI and smoking status were derived from the most recent entry in CPRD-GOLD. Socioeconomic status was measured using the Index of Multiple Deprivation (IMD) which was categorised into deciles (Q1 = least deprived; Q10 = most deprived). Deprivation scores were available only for linked patients (*N* = 16,767). In cases where a patient’s deprivation score was missing, the practice’s deprivation score was used to replace the missing value (in total *N* = 16,851). Ethnicity was classified into two groups, White or Black, Asian and Minority Ethnic (BAME), using both CPRD-GOLD and Admitted Patient Care Hospital Episodes Statistics.

Patients were followed up for two years in order to assess the following healthcare service utilisation and mortality outcomes: (i) primary care consultations per year, (ii) new prescriptions issued per year; (iii) all-type hospitalisation admissions per year; and (iv) the 2-year mortality rate [44]. Primary care consultations included any phone or in-person contact with a clinician in primary care. Hospital admissions were identified from discharge dates in Hospital Episodes Statistics (HES) dataset. Data linkage procedures are described in detail in Additional file 2: Figure S1.

### Statistical analysis

First, we investigated the co-occurrence of the 36 LTCs in primary care records by calculating the overall prevalence of multimorbidity and its distribution across demographic group. Next, we calculated the prevalence of each condition in the entire sample and by age group, using chi-square tests to statistically assess differences between age groups.

To identify clusters of co-morbidities within each age group, we applied latent class analysis (LCA), a model-based clustering approach that calculates class membership probabilities among stroke/TIA patients, and assigns patients to a single, non-overlapping cluster -corresponding to the latent class with the highest class membership probability- based on similar patterns of morbidities.

The optimal number of clusters was determined by reviewing model fit indices (Bayesian Information Criterion (BIC), sample-sized adjusted BIC, Akaike Information Criterion (AIC), Consistent AIC, log-likelihood ratio test, and entropy for classification quality), combined with clinical interpretability. To ensure meaningful class sizes, the number of generated classes was limited to a maximum of 10, consistent with previous research [48]. Given the skewed distribution of comorbidities—where certain combinations are highly prevalent while others are rare- we only included conditions with prevalence ≥1% in the entire sample. This threshold was the lowest cutoff that avoided model convergence issues (negative degrees of freedom) while preserving clinically meaningful conditions.

The final model was used to characterise and label the clusters based on comorbidities and outcome patterns. Each cluster was further described in terms of demographic characteristics (gender, age, deprivation, smoking status, BMI), healthcare utilisation and mortality. Categorical data were presented as percentages, while continuous data were summarised using median and interquartile range (IQR).

To explore associations between key demographic characteristics, health outcomes (mortality, hospital admission, primary care appointments, medication prescription) and clusters, we used generalised linear models. Negative binomial models were employed to analyse the number of GP consultations, prescriptions, new prescriptions, and hospital admissions over a 2-year period, adjusting for gender, deprivation, smoking status, BMI and age. Incidence rate ratios (IRR) with 95% confidence intervals were estimated. For 2-year mortality, we used logistic regression models with the same demographics adjustments, reporting odds ratio (ORs) and 95% confidence intervals. The cluster of lower morbidity and better outcomes was used as a reference group in all of these models.

The LCA included patients with recorded co-morbidities or functional impairments, regardless of the missing data in other variables. Complete case analysis using pairwise deletion was applied for descriptive comparisons. The analysis of the hospitalisation outcomes was limited to patients linked to HES. As an unsupervised method, LCA identifies patterns of multimorbidity and does not establish causal relationships; therefore, associations between clusters and outcomes were interpreted as descriptive rather than explanatory.

### Assessment of clusters’ robustness

We assessed the robustness of the clustering solution, in two tiers. First, we randomly split the dataset into training (80% of the stroke/TIA patients) and test (20%) subsets. LCA was initially performed on the training set to determine the optimal number of clusters per age group which was used for the performance of the method on the test set. In this way, we checked the consistency between the clusters in two independent datasets (the training and the test set) in terms of the most prevalent conditions.

To further investigate the stability of the clusters we calculated the Pearson correlation coefficients between the corresponding clusters in the two subsets, matching them based on condition prevalence. Values closest to 1 demonstrate the strongest the association between clusters.

More details on the method used are provided in Additional File 1. All data management and statistical analyses was conducted in R and STATA/SE v.18.0.

### Role of the funding source

The funder of the study had no role in study design, data collection, data analysis, data interpretation, or writing of the report.

## Results

### Demographic characteristics of the study population

The analysis included 69,372 eligible adults (Additional file 2: Fig. S1) from England (31.56%), Scotland (36.31%) and Wales (25.44%). The majority of the stroke/TIA survivors were aged 65 years or older (*N* = 53,748; 77.48%), and the mean BMI indicated that the cohort was, on average, overweight (M_BMI_= 27.8$\:\pm\:$5.64)). 47% (*N* = 32,603) had never smoked, while ethnicity data were available for 57.3% (*N* = 39,774) of the patients, of whom 89.2% were white (*N* = 35,498; representing 51.17% of the overall sample). Socioeconomic deprivation data were available for English patients only (24.3% of the overall sample, *N* = 16,851 patients), of whom 15.92% lived in the least deprived decile, and 7.23% (*N* = 1,219) in the most deprived decile (Table 1 and Additional file 2: Table S3).

### Prevalence of multimorbidity

Most stroke/TIA survivors (*N* = 65,687; 94.69%) experienced at least one additional LTC. The prevalence of multimorbidity increased with age, from 67.41% (*N* = 1,059) among those aged 18–44 to 99.15% (*N* = 13,799), in individuals older than 85 years (Table 1). Multimorbidity was also more prevalent among people living in the most deprived areas (*N* = 1,164; 95.49%) (Additional file 2: Table S3).

**Table 1:** Demographic characteristics of the study population and multimorbid patients

	Whole sample *N*(%)	Multimorbid patients (%)*	Total number of LTCs				No. of LTCs, median [IQR]
			Only stroke/ TIA; *N* (%)	2–4 *N* (%)	5–7; *N* (%)	> 7 *N* (%)	
All patients	69,372 (100)	65,687 (94.69)	3,685 (5.31)	29,375 (42.34)	26,243 (37.83)	10,070 (14.52)	5 [3–6]
Gender							
Male	36,189 (52.17)	33,979 (93.89)	2,210 (6.11)	16,331 (45.13)	12,932 (35.73)	4,716 (13.03)	4 [3–6]
Female	33,183 (47.83)	31,708 (95.55)	1,475 (4.45)	13,044 (39.31)	13,310 (40.11)	5,354 (16.13)	5 [3–7]
Age							
[18–44]	1,571 (2.26)	1,059 (67.41)	512 (32.59)	859 (54.68)	174 (11.08)	26 (1.65)	2 [1–3]
[45–64]	14,053 (20.26)	12,365 (87.99)	1,688 (12.01)	7,711 (54.87)	3,733 (26.56)	921 (6.55)	3 [2–5]
[65–84]	39,831 (57.42)	38,464 (96.57)	1,367 (3.43)	16,976 (42.62)	15,622 (39.22)	5,866 (14.73)	5 [3–6]
[85+]	13,917 (20.06)	13,799 (99.15)	118 (0.85)	3,829 (27.51)	6,713 (48.24)	3,257 (23.40)	6 [4–7]
Ethnicity†							
BAME	4,276 (6.16)	4,032 (94.29)	244 (5.71)	1,889 (44.18)	1,533 (35.85)	610 (14.27)	5 [3–6]
White	35,498 (51.17)	33,679 (94.88)	1,819 (5.12)	14,595 (41.11)	13,734 (38.69)	5,350 (15.07)	5 [3–6]

**Notes**: *: percentages calculated over the demographic group

†: For 29,598 patients (42.67% of the sample) the ethnicity was unknown

The number of LTCs per patient ranged from 1 (i.e., only stroke/TIA) to 7, with a mean of 4.91 (SD = 2.41) and a median of 5 (IQR, 3 to 6) (Table 1). Hypertension (59.65%), painful conditions (38.44%), and coronary heart disease (21.99%) were the most prevalent comorbidities. The prevalence of each LTC by age group is presented in Additional file 2: Table S4.

**Fig. 1:**
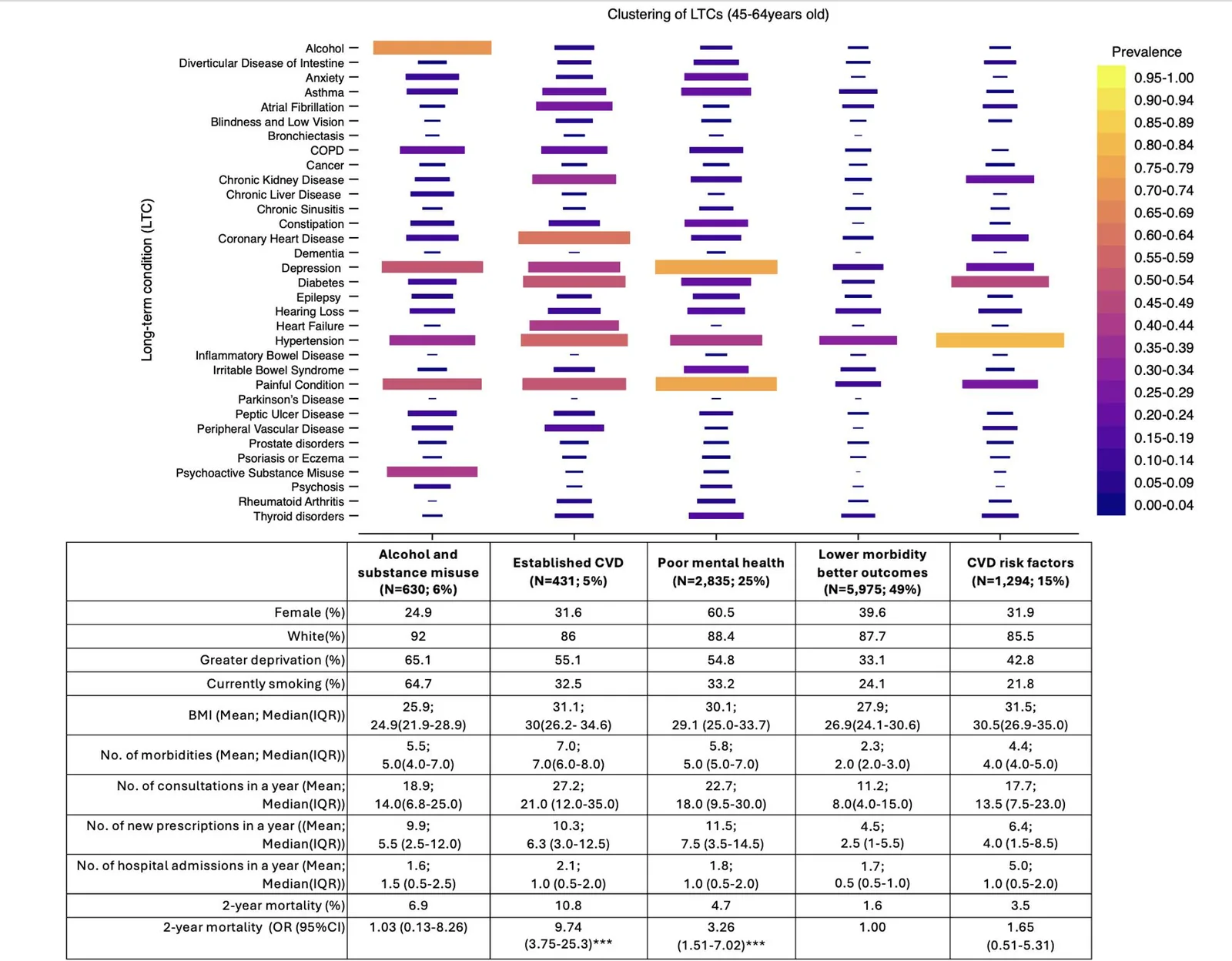
Clustering of multimorbidity in stroke survivors 45-64 years old. Notes: The upper panel displays the prevalence of long-term conditions within each latent class, with colour intensity representing prevalence (from low [dark] to high [yellow]). Each column corresponds to a latent class, and each row to a specific condition. The lower panel summarises demographic characteristics and outcomes ( number of consultations in a year, number of new prescriptions in a year, number of hospital admissions in a year, 2-year mortality) across classes. Mortality was analysed using regression models as the primary outcome. Regression analyses for other outcomes are presented in Additional file 2: Table S13. The 2-year mortality logistic regression model was adjusted for gender, deprivation, smoking status, BMI and age. *** *p* < 0.001

### Multimorbidity clusters and outcomes

**Fig. 2:**
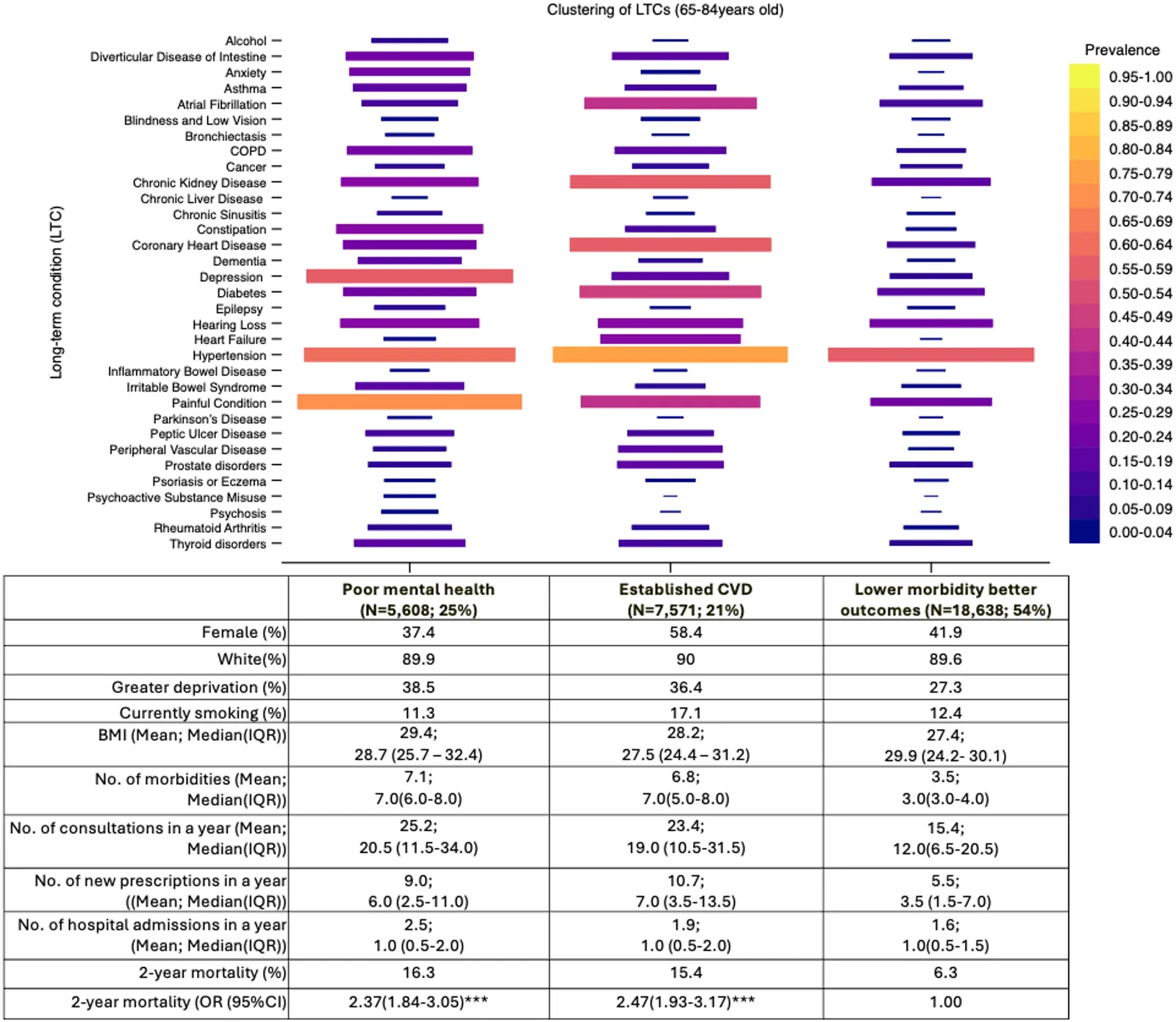
Clustering of multimorbidity in stroke survivors 65–84 years old. Notes: The upper panel displays the prevalence of long-term conditions within each latent class, with colour intensity representing prevalence (from low [dark] to high [yellow]). Each column corresponds to a latent class, and each row to a specific condition. The lower panel summarises demographic characteristics and outcomes ( number of consultations in a year, number of new prescriptions in a year, number of hospital admissions in a year, 2-year mortality) across classes. Mortality was analysed using regression models as the primary outcome. Regression analyses for other outcomes are presented in Additional file 2: Table S14. The 2-year mortality logistic regression model was adjusted for gender, deprivation, smoking status, BMI and age. *** *p* < 0.001

**Fig. 3:**
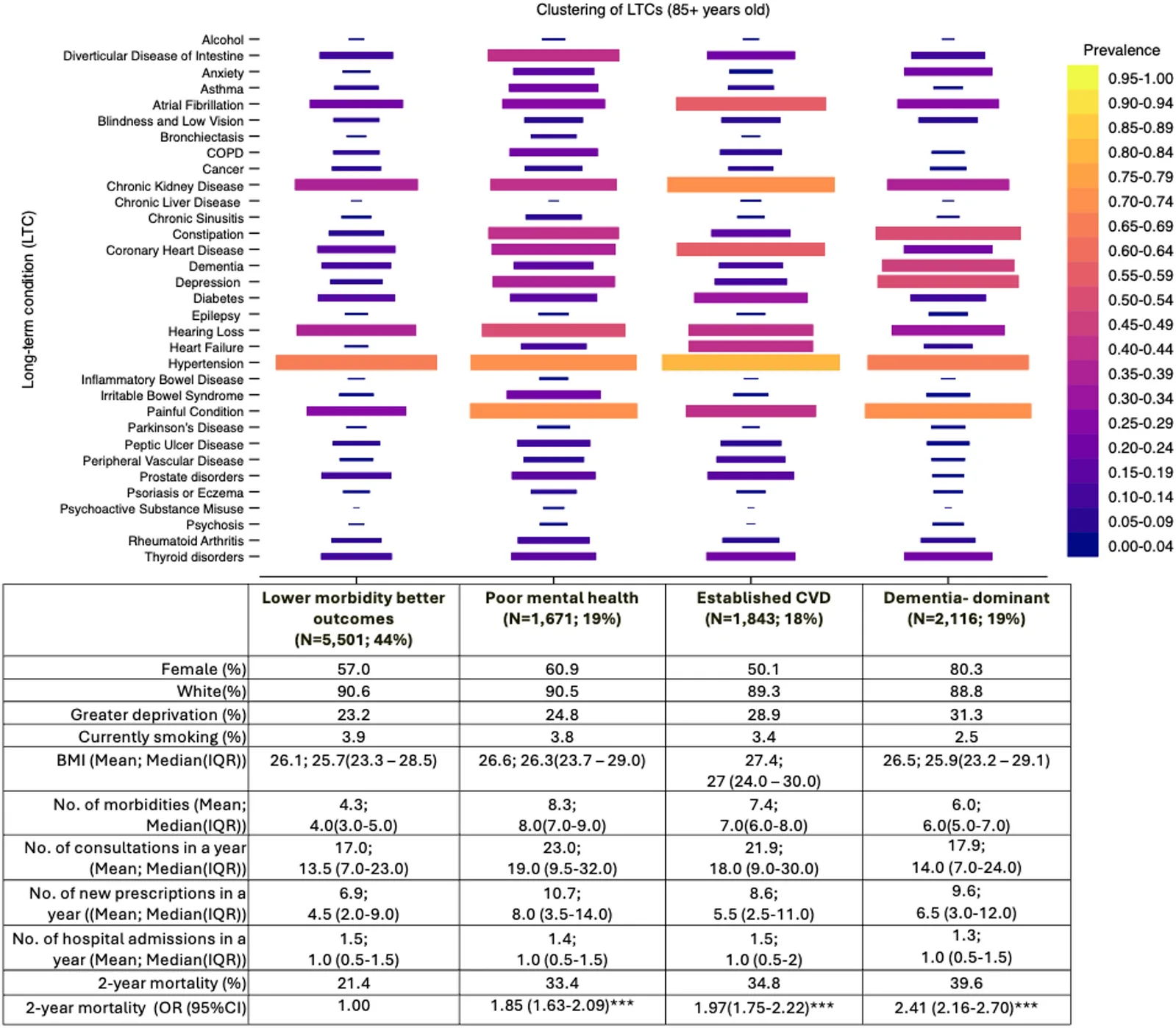
Clustering of multimorbidity in stroke survivors over 85 years old. Notes: The upper panel displays the prevalence of long-term conditions within each latent class, with colour intensity representing prevalence (from low [dark] to high [yellow]). Each column corresponds to a latent class, and each row to a specific condition. The lower panel summarises demographic characteristics and outcomes ( number of consultations in a year, number of new prescriptions in a year, number of hospital admissions in a year, 2-year mortality) across classes. Mortality was analysed using regression models as the primary outcome. Regression analyses for other outcomes are presented in Additional file 2: Table S15. The 2-year mortality logistic regression model was adjusted for gender, deprivation, smoking status, BMI and age. *** *p* < 0.001

We identified five clusters among patients aged 45–64 (Fig. 1), three clusters among patients aged 65–84 (Fig. 2) and four clusters among patients aged 85 and over (Fig. 3). For ease of reference, we have subjectively chosen a name for each cluster based upon particularly prevalent LTCs or notable outcomes or demographic features within that cluster. The clustering results of the 2.26% of our population that was under the age of 45 and had lower rates of multimorbidity, are presented in Additional file 2: Fig. S2a, S3, and Tables S5, S6, S10- S12.

### Age strata: 45–64 years old

A cluster characterised by relatively high prevalence of alcohol and substance misuse, and a cluster with the highest levels of established cardiovascular disease (including atrial fibrillation, chronic kidney disease, coronary heart disease, heart failure and peripheral vascular disease) account for just 6% and 5% of patients within this age stratum respectively but have the highest rates of mortality. The “alcohol and substance misuse” cluster is more male, white, deprived and has highest smoking rates, while adjustment for these demographic factors demonstrates that they account for the association with increased mortality. By contrast, in the “established CVD” cluster, the association with increased mortality remains after adjustment for demographic factors (Fig. 1).

A third, “poor mental health”, cluster has the highest prevalence of depression, anxiety, IBS and painful conditions, and accounts for 25% of patients in this age strata. A fourth “lower morbidity, better outcomes” cluster with a mean of just 2.3 co-morbidities per patient accounts for the largest proportion of patients (49%) in this age strata, and also exhibits the lowest rates of mortality, prescriptions and consultations. A fifth “CVD risk factors” cluster had the highest prevalence of hypertension but had lower rates of established cardiovascular disease and better clinical outcomes when compared to the rarer “established CVD” cluster.

### Age strata: 65–84 years old

In the age 65–84 age stratum, LCA revealed three clusters (Fig. 2). First, there is again a “poor mental health” cluster characterised by the highest levels of depression and painful conditions. Second, there is again an “established CVD” cluster, characterised by the highest levels of established cardiovascular diseases.

Finally, there is again a cluster that can be described as “lower morbidity, better outcomes”, accounting for 54% of patients in this age strata, alongside the lowest rates of co-morbidity (mean of 3.5), fewer consultations, fewer prescriptions, fewer hospital admissions and lower mortality versus the other two clusters identified in the age 65–84 age strata.

### Age strata: 85 and older

In the 85 + age stratum, LCA showed four clusters (Fig. 3). Again, there were clusters that could be characterised by “lower morbidity and better outcomes”, “poor mental health”, and “established CVD”.

A “lower morbidity, better outcomes” cluster was again most prevalent (accounting for 44% of patients in the age strata) and had the lowest rates of co-morbidity, consultations, prescriptions and mortality. A “poor mental health” cluster again has the second highest rates of depression, chronic pain and anxiety. A “established CVD” cluster had the highest prevalence of all clusters of atrial fibrillation, chronic kidney disease, coronary heart disease, diabetes, heart failure, hypertension and peripheral vascular disease.

It is the fourth cluster in this age stratum with “dementia-dominant”, accounting for 19% of patients, that has the highest 2-year mortality of all the clusters in this age stratum (39.6%). While this cluster is most notable for its markedly raised prevalence of dementia (45.3%), it also contains the highest prevalence of depression (51.6%) and has a preponderance of females (80.3%).

### Validation of the clustering models

For the age stratum 45–64, the validation process indicated a good degree of concordance between the training- and the test-derived clusters. The training-derived clusters of “alcohol and substance misuse”, “poor mental health”, “lower multimorbidity better outcomes” and “CVD risk factors” indicated high replicability and had strong positive Pearson’s correlation coefficients > 0.97 with the corresponding test-derived cluster. However, the “established CVD” cluster had a notably weaker correlation with its closest test-derived cluster (0.86), and more closely resembled the similar “CVD risk factors” cluster (Additional file 2: Table S11). This reduced concordance likely reflects real life clinical heterogeneity: cardiovascular disease in middle-aged stroke survivors spans a heterogeneous spectrum, ranging from early cardiometabolic risk factors to established cardiovascular disease, with varying combinations and severity, which leads to overlapping multimorbidity patterns that are difficult to separate into fully distinct latent classes. From a modelling perspective, this apparent instability is consistent with boundary overlap between closely related cardiovascular profiles rather than with a failure of the clustering approach.

The clusters derived by the training set of the patients aged 65–84 all demonstrated high (> 0.99) Pearson’s correlation coefficients with distinct corresponding test-derived clusters. Likewise, the clusters of multimorbidity identified among stroke/TIA survivors over 85 years old also demonstrated high (> 0.95) Pearson’s correlation coefficients with distinct corresponding test-derived clusters. The details of these findings are given in Additional file 2: Tables S10 and S11.

## Discussion

### Summary of results and comparison with other studies

This study is the first to identify and validate clusters of stroke survivors with multimorbidity and is consistent with previous research in demonstrating that 95% of UK adult stroke/TIA survivors live with multimorbidity [3, 17]. Each cluster had distinct patterns of LTCs and different healthcare needs and outcomes. To take one example, among 45–64-year-olds, an “established cardiovascular disease” cluster comprised just 5% of patients within this age strata yet had the highest rate of co-morbidities (mean 7.0) and mortality (10.8% over 2 years). By contrast, a “lower morbidity, better outcomes” cluster included half of patients and had vastly better outcomes (mortality of 1.6% over 2 years). Such contrasts between clusters highlight the varied needs of different types of stroke patients.

Across the three age strata we present, a mental-health-related cluster with high rates of chronic pain and depression was consistently observed, highlighting that these common conditions are present and important in many stroke survivors too. In the oldest age strata only, a dementia-dominant cluster was identified, and this cluster had the highest mortality rate. More positively, each age strata included over 40% of patients in a lower morbidity and better outcomes cluster.

One previous study also identified patterns of multimorbidity associated with stroke, using the timing and sequencing of LTCs over the life course [35]. Their nine clusters included clusters characterised by cardiometabolic conditions, and mental health conditions as well as low-morbidity clusters typically associated with younger age; findings that mirror the structures identified in our age-stratified analysis. However, because the study was not age-stratified, co-morbidity patterns in clusters were largely reflective of their age profile. The study was also smaller, unvalidated, and based on a London population of stroke survivors.

When compared with existing LCA studies in general elderly populations, our findings indicate that the identified clusters include patterns of multimorbidity, such as the co-occurrence of neurological, vascular, and functional conditions [41]. For example, although dementia is observed in general older populations, its prominence and combination with stroke-related comorbidities in our study are more consistent with post-stroke trajectories.

### Strengths and limitations

The identification of clusters of multimorbidity has been based on data representative of the UK population in terms of age, sex and ethnicity [19]. Unlike studies that have used disease-centred approaches for the investigation of multimorbidity, our approach is a patient-centred approach that assigns each patient into a specific cluster, regardless of the number of conditions they suffer, while the identification of the number of clusters has been based on a conjunction of statistical criteria, clustering interpretation and clinical input. Unlike the recent study of Delord et al. [35] that aimed to identify longitudinal patterns of multimorbidity, our approach preserves heterogeneity that would otherwise be diluted in a single, population-wide clustering solution. This allows clinically relevant patterns—such as mental-health–dominant profiles in mid-life or dementia-driven multimorbidity in the oldest group—to emerge clearly and without competing with younger-onset conditions. While trajectory-based clustering approaches offer deeper insights into the temporal evolution of multimorbidity, the LCA approach used here provides a pragmatic, outcomes-oriented structure that aligns closely with the needs of clinicians, and policymakers tasked with organising stroke aftercare. While there is no consensus definition of what should be included in a reasonably comprehensive list of LTCs, we used a list that has been used widely in primary care research, including both physical and mental health LTCs to permit broad investigation of patients’ needs and outcomes [5, 46]. Two-tier validation of clusters ensured replicability. Our methodological approach has been used successfully before, providing further support for the robustness and the consistency of our approach [39, 41].

The validation process indicated that the patterns of multimorbidity were robust and internally validated, with consistent disease co-occurrence across independent samples in 11 out of the 12 clusters identified.

This study has some limitations. First, the use of routinely collected data can be inaccurate due to misclassification, variability in coding practices across GPs or incomplete recording. Previous validation studies of CPRD data have shown that recording accuracy varies by condition, with positive predictive values typically ranging from 80 to 95% for major chronic diseases such as diabetes and diagnosed hypertension, but lower for mental health conditions, musculoskeletal conditions, irritable bowel syndrome and lifestyle-related factors [19, 24].

This can lead to underestimation of the prevalence of the condition, misclassification of multimorbidity cluster or inaccurate description of the cluster. Furthermore, differential recording practices, where individuals with one diagnosed condition (e.g., diabetes) are more likely to be screened and recorded for others (e.g., hypertension), could introduce bias linked to healthcare utilisation or demographic factors such as age, gender, and socioeconomic status.

Second, we focused on multimorbidity of people with a previous stroke/TIA who could be identified in primary care records which, despite incentivisation to maintain accurate stroke/TIA survivor registers through the UK’s Quality and Outcomes Framework (QOF), will miss some patients [24].

Third, ethnicity was dichotomised into “White” and “BAME,” with substantial missingness (~ 43%). This approach may obscure meaningful differences between diverse ethnic groups and limits the ability to fully explore inequalities in multimorbidity patterns and outcomes.

Further, while UK primary care is available free to all UK residents regardless of nationality, some populations, such as undocumented migrants, may avoid all contact with the formal health sector. Our study population was also a prevalent stroke population, so under-represents stroke survivors with poor survival or who have recently moved practice (e.g. as a result of moving to a nursing home).

### Implications for policy, practice, research

These results may inform new approached to organising health services for stroke survivors (see Table 2 for authors’ proposed recommendations). Each cluster that we identified represents a potential target population for the application (and testing) of new health care models. For example, the largest proportion of stroke survivors at all ages have lower levels of multimorbidity and better outcomes. It is conceivable that most of their long-term healthcare needs could be largely managed in primary care and with self-care: innovation could focus on meeting such needs with greater efficiency, thus freeing up resources to better support more complex patients. By contrast, a smaller proportion of stroke survivors at all ages have particularly poor mental health, with high levels of depression and painful conditions, highlighting the need to better integrate mental and physical health care in these patients.

Current guidelines for stroke care do not take into account the nuance that, with regards to longer term care, different approaches might apply to different clusters. For example, the UK guidelines recommend that holistic reviews are carried out at regular intervals for all stroke survivors [49]. This failure to differentiate is one of the possible explanations for the failure of a primary care based model of stroke after care to achieve any measurable benefit in a randomised controlled trial [50].

**Table 2:** Specific practice and policy implications for each cluster

45–64 year old cluster types (% of patients)	Alcohol and substance misuse (6%)	Established CVD (5%)	Poor mental health (25%)	Lower morbidity and better outcomes (49%)	CVD risk factors (15%)
Practice and policy implications	Social work teams work with a named general practitioner to address complex social problems.	A single generalist “cardio-metabolic” physician coordinates hospital care between specialities.	Social prescribers manage the determinants and effects of both physical and mental health problems. Social prescribers are provided with stroke related resources and training. Bespoke psychological therapy is tailored to long term conditions	Digital health provide patients with the understanding and control to manage their condition and seek stepped up support when required. For example, patients are trained to access the NHS app alongside secondary prevention monitoring and stroke support resources.	Clinical pharmacists are assigned to each patient to support medication adherence, aim for strict secondary prevention targets, and manage poly-pharmacy.
65–84 year old cluster types (% of patients)		Established CVD (21%)	Poor mental health (25%)	Lower morbidity and better outcomes (54%)	
Practice and policy implications		*As above*	*As above*	*As above*	
85 + year old cluster types (% of patients)	Dementia-dominant (19%)	Established CVD (18%)	Poor mental health (19%)	Lower morbidity and better outcomes (44%)	
Practice and policy implications	Older people’s social work teams work with a named general practitioner to support the patient and their carer.	Geriatricians may now be best place to coordinate hospital care.	*As above*,* adjusted for age*	*As above*,* adjusted for age*	

Further research might seek to replicate our findings in a non-UK population, and to test novel interventions in groups of patients that resemble the clusters we have identified. Additionally, improved recording of frailty, disability and psychosocial factors in routine healthcare data is required to provide a broader understanding of the needs of stroke patients than is possible with current data.

## Conclusions

In conclusion, our study has identified and validated clusters of stroke survivors can with distinct patterns of multimorbidity, healthcare utilisation, and mortality rates. Understanding these clusters provides valuable insights for developing targeted healthcare services and interventions for stroke survivors.

## Supplementary Information

Below is the link to the electronic supplementary material.

Supplementary Material 1: Additional file 1. Tables S1- S2. Table S1 - Clinical codes of stroke. Table S2 - Long-term conditions’ definition.

Supplementary Material 2: Additional file 2. Figure S1, S2a- S2d, 3. Tables S3-S15.

## Data Availability

The study uses data from the CPRD. CPRD does not allow the sharing of patient-level data. The data specification for the CPRD data set is available at: [https://www.cprd.com/data/primary-care-data/cprd-gold](https:/www.cprd.com/data/primary-care-data/cprd-gold) . Code lists are shared in our supplementary. Analysis code is available upon request.

## Abbreviations

TIA: Transient Ischaemic Attack
LTCs: Long-Term Conditions
GP: General Practitioner
CPRD: Clinical Practice Research Datalink
LCA: Latent Class Analysis
IMD: Index of Multiple Deprivation
BMI: Body Mass Index
BAME: Black, Asian and Minority Ethnic
HES: Hospital Episodes Statistics
BIC: Bayesian Information Criterion
AIC: Akaike Information Criterion
IQR: interquartile Range
IRR: Incidence rate ratios
