# Supplementary material for: Clustering of multimorbidity in stroke and transient ischaemic attack survivors: a population-based study
Source: BMC Med. 2026 May 18;24:472. doi: 10.1186/s12916-026-04924-7 (PMC13548555; doi:10.1186/s12916-026-04924-7)
Supplement: Supplementary file 1 [file 12916_2026_4924_MOESM1_ESM.docx]

**Additional file 1**

Correspondence to: Dr Efthalia Massou; em716@medschl.cam.ac.uk

This additional file goes along with the main paper and includes methodological details.

In this study, stroke cases were identified using Read Codes (Table S1). Read codes are codes used by GPs on computerised records to indicate diagnoses, symptoms, and other clinical activities [21].

[Table S1 here]

We defined multimorbidity as the occurrence of two or more of 36 long-term conditions (LTCs) recorded in a patient’s primary care medical records. The list of these 36 conditions (available in Table S2) includes chronic conditions that significantly affect quality of life, healthcare utilisation, morbidity and mortality. This list has been widely used in UK primary care electronic health records research [5,22].

[Table S2 here]

*Statistical analysis*

In this paper, socioeconomic status was measured using the Index of Multiple Deprivation (IMD), the official measure of relative deprivation in England, which consists of seven domains: income; employment; health and disability; education, skills, and training; barriers to housing and services; crime; and living environment [33]. Deprivation scores were available only for linked patients. In cases where patient-level deprivation was missing, practice-level deprivation was used as a proxy, consistent with previous CPRD studies [34].

.

*Latent class analysis*

Various analytical approaches have been used in literature to explore multimorbidity patterns. These include distance-based clustering techniques such as k-means and its variants, unsupervised patient-oriented clustering based on the Ward’s minimum variance criterion and multiple state clustering analysis (MSCA) [35-38]. While these approaches have provided valuable insights into multimorbidity patterns, they often rely on predefined distance metrics or deterministic cluster assignments, or they lack validation of the derived clusters.

To address these methodological limitations and identify clusters of co-morbidity within each age group of stroke/TIA survivors, we applied latent class analysis (LCA). Previous studies have shown that LCA tends to produce the most consistent and interpretable multimorbidity clusters that has the closest agreement with known disease groupings [39]. The method is a cross-sectional latent variable mixture modelling (LVMM) approach that leverages observed variables to uncover unobserved heterogeneity within the population of interest. LCA relies on probabilistic modelling to identify latent classes within a heterogeneous population, which differ in their underlying characteristics and are distinct from each other. This model-based clustering analysis calculates the probability of belonging to each latent class for each individual, assigning stroke/TIA survivors to latent classes in a person-centred rather than a disease-centred approach. This method allowed us to assign each patient to a single, non-overlapping cluster -corresponding to the latent class with the highest-class membership probability- based on similar patterns of morbidities. In this way, LCA offers the advantage of identifying clusters that are less sensitive to subjective choices regarding the number of clusters or the distance measures used.

Given the skewed distribution of comorbidities - where certain combinations are highly prevalent while others are rare – and in order to address the issue of negative degrees of freedom, we limited the conditions included in the LCA. Specifically, we only included conditions with prevalence equal or higher than 1% in the entire sample. This threshold was determined pragmatically through preliminary analyses since including conditions below this cutoff resulted in model convergence issues, particularly in age-stratified models with smaller sample sizes.

*Model selection statistics*

The optimal number of clusters in each age group, was determined by reviewing diagnostic statistics including the Bayesian Information Criterion (BIC), the sample-sized adjusted BIC, the Akaike Information Criterion (AIC), the adjusted AIC, the log-likelihood, the likelihood ratio test, and entropy for classification quality. These model fit indices were calculated based on how well each solution (varying number of classes) reproduced the observed joint distribution of all included comorbidity indicators [40]. The Bayesian Information Criterion (BIC) and the Sample-size Adjusted BIC (SABIC) are regarded as the most reliable and consistent indicators of the optimal number of clusters, particularly when categorical variables were used in clustering methodology. Since overfitting is a major risk in clustering when adding more clusters, this property is particularly useful in LCA. Additionally, SABIC modifies BIC to account for smaller sample sizes, making it more appropriate when the dataset is not extremely large. A lower BIC value indicates a better balance between number of clusters and model parsimony and is preferred over the competing solutions. AIC and its adjusted version that corrects AIC for small sample sizes, follow the same principle, where lower values indicate better cluster solutions. However, AIC and adjusted AIC may favour more complex models often selecting the solution with the greater number of clusters. In LCA where choosing too many clusters can lead to poor interpretability, AIC might perform poorly compared to BIC, since the latter penalises model complexity more heavily than the former. On the other hand, entropy measures the separation quality of clusters, and although there is no universally accepted threshold, values above 0.8 are considered acceptable and while values close to 1 indicate an ideal solution. In terms of the log-likelihood, higher values indicate a better fit, while in terms of the log-likelihood ratio that compares two nested models, lower values indicate better model performance. However, this statistic measures only the model fit of the data and does not penalise complexity. These criteria were assessed alongside clustering interpretability and clinical input. To ensure meaningful class sizes, the number of generated classes was limited to a maximum of 10, consistent with previous research that using general population suggested that more than 10 classes result in class sizes of < 1%.

*Validation of the latent class solution*

To assess the validity and reliability of morbidity cluster, we randomly divided our sample into two subsets: a training set (80% of the stroke/TIA patients) and a test set (20%). LCA was initially performed on the training set to determine the optimal number of clusters per age group. The analysis was then repeated on the test set using the predefined number of clusters from the training phase. Our aim was to identify in the test set, each cluster identified in the training test. In this way, we checked the consistency between the clusters in the training and the test sets in terms of the most prevalent conditions.

To further investigate the stability and robustness of the clustering solutions, we calculated the Pearson correlation coefficients between the corresponding clusters in the two subsets, matching them based on condition prevalence. The closer to 1 was the values, the strongest the association between the cluster. Our methodological approach followed the framework developed by Zhu et al using the general population and has been applied in other similar studies [41-43].

*Description of the clusters*

Patients were followed up for two years in order to assess the following healthcare service utilisation and mortality outcomes: i) primary care consultations per year, ii) new prescriptions issued per year; iii) all-type hospitalisation admissions per year; and iv) the 2-year mortality rate [44-45]. Primary care consultations included any phone or in-person contact with a clinician in primary care. Hospital admissions were identified from discharge dates in Hospital Episodes Statistics (HES) dataset.

Once the best model was selected, we characterised and labelled the clusters in terms of comorbidities and outcome patterns. Each cluster was further described in terms of demographic characteristics (gender, age, deprivation, smoking status, BMI) and healthcare utilisation and mortality rates. Categorical data were presented as percentages, while continuous variables were summarised using median and interquartile range (IQR).

To explore associations between key demographic characteristics, health outcomes (mortality, hospital admission, primary care appointments, medication prescription) and clusters, we used generalised linear models. Negative binomial models were employed to analyse the number of GP consultations, prescriptions, new prescriptions, and hospital admissions over a 2-year period, adjusting for gender, deprivation, smoking status, BMI and age. Incidence rate ratios (IRR) with 95% confidence intervals were estimated. For 2-year mortality, we used logistic regression models with the same demographics adjustments, reporting odds ratio (ORs) and 95% confidence intervals. We acknowledge that Cox proportional hazards models could be used where complete time-to-event data are available, and this represents a limitation of this analysis and a potential extension for future work. However, our primary objective for mortality was to estimate the association between multimorbidity clusters and the risk of death within a fixed 2-year follow-up period, rather than to model time-to-death. In addition, precise dates of death and consistent start times for follow-up were not uniformly available for all patients, which limited the reliable implementation of time-to-event models and promote the use of logistic regression.

The LCA included patients with recorded co-morbidities and functional impairments, regardless of the missing data in other variables. To describe clusters in terms of the demographics, primary and secondary healthcare utilisation, and mortality we conducted complete case analysis using pairwise deletion. Hospitalisation outcomes analysis was limited to patients linked to HES.

**Table S1. Clinical codes of stroke**

| **Medcode** | **Readcode** | **Description** | **Coding System** |
| --- | --- | --- | --- |
| 504 | G65..00 | Transient cerebral ischaemia | Read Code |
| 569 | G64..12 | Infarction - cerebral | Read Code |
| 1298 | G66..11 | CVA unspecified | Read Code |
| 1433 | G65..12 | Transient ischaemic attack | Read Code |
| 1469 | G66..00 | Stroke and cerebrovascular accident unspecified | Read Code |
| 1895 | G65z.00 | Transient cerebral ischaemia NOS | Read Code |
| 2418 | G6...00 | Cerebrovascular disease | Read Code |
| 3149 | G64z.00 | Cerebral infarction NOS | Read Code |
| 3535 | G61z.00 | Intracerebral haemorrhage NOS | Read Code |
| 5051 | G61..00 | Intracerebral haemorrhage | Read Code |
| 5185 | G64z111 | Lateral medullary syndrome | Read Code |
| 5363 | G64..11 | CVA - cerebral artery occlusion | Read Code |
| 5602 | G64z.12 | Cerebellar infarction | Read Code |
| 6116 | G66..13 | CVA - Cerebrovascular accident unspecified | Read Code |
| 6155 | G64..13 | Stroke due to cerebral arterial occlusion | Read Code |
| 6253 | G66..12 | Stroke unspecified | Read Code |
| 6960 | G61..11 | CVA - cerebrovascular accid due to intracerebral haemorrhage | Read Code |
| 7780 | G667.00 | Left sided CVA | Read Code |
| 7912 | G614.00 | Pontine haemorrhage | Read Code |
| 8443 | G663.00 | Brain stem stroke syndrome | Read Code |
| 8837 | G64..00 | Cerebral arterial occlusion | Read Code |
| 9985 | G64z200 | Left sided cerebral infarction | Read Code |
| 10504 | G64z300 | Right sided cerebral infarction | Read Code |
| 12833 | G668.00 | Right sided CVA | Read Code |
| 13564 | G613.00 | Cerebellar haemorrhage | Read Code |
| 15019 | G641.00 | Cerebral embolism | Read Code |
| 15252 | G64z.11 | Brainstem infarction NOS | Read Code |
| 15788 | G65zz00 | Transient cerebral ischaemia NOS | Read Code |
| 16507 | G65z100 | Intermittent cerebral ischaemia | Read Code |
| 16517 | G640.00 | Cerebral thrombosis | Read Code |
| 16956 | G669.00 | Cerebral palsy, not congenital or infantile, acute | Read Code |
| 17322 | G664.00 | Cerebellar stroke syndrome | Read Code |
| 18604 | G61..12 | Stroke due to intracerebral haemorrhage | Read Code |
| 18689 | G660.00 | Middle cerebral artery syndrome | Read Code |
| 19201 | G61X100 | Right sided intracerebral haemorrhage, unspecified | Read Code |
| 19260 | G662.00 | Posterior cerebral artery syndrome | Read Code |
| 19280 | G661.00 | Anterior cerebral artery syndrome | Read Code |
| 19354 | G65y.00 | Other transient cerebral ischaemia | Read Code |
| 23671 | G63y000 | Cerebral infarct due to thrombosis of precerebral arteries | Read Code |
| 24446 | G63y100 | Cerebral infarction due to embolism of precerebral arteries | Read Code |
| 25615 | G64z000 | Brainstem infarction | Read Code |
| 26424 | G64z400 | Infarction of basal ganglia | Read Code |
| 27975 | G641000 | Cerebral infarction due to embolism of cerebral arteries | Read Code |
| 28314 | G61X000 | Left sided intracerebral haemorrhage, unspecified | Read Code |
| 30045 | G616.00 | External capsule haemorrhage | Read Code |
| 30202 | G617.00 | Intracerebral haemorrhage, intraventricular | Read Code |
| 31060 | G61X.00 | Intracerebral haemorrhage in hemisphere, unspecified | Read Code |
| 31595 | G610.00 | Cortical haemorrhage | Read Code |
| 33499 | G665.00 | Pure motor lacunar syndrome | Read Code |
| 33543 | G6X..00 | Cerebrl infarctn due/unspcf occlusn or sten/cerebrl artrs | Read Code |
| 34758 | G641.11 | Cerebral embolus | Read Code |
| 36717 | G640000 | Cerebral infarction due to thrombosis of cerebral arteries | Read Code |
| 39344 | G676000 | Cereb infarct due cerebral venous thrombosis, nonpyogenic | Read Code |
| 40338 | G611.00 | Internal capsule haemorrhage | Read Code |
| 40758 | G6W..00 | Cereb infarct due unsp occlus/stenos precerebr arteries | Read Code |
| 44765 | G653.00 | Carotid artery syndrome hemispheric | Read Code |
| 46316 | G612.00 | Basal nucleus haemorrhage | Read Code |
| 47642 | G64z100 | Wallenberg syndrome | Read Code |
| 50594 | G654.00 | Multiple and bilateral precerebral artery syndromes | Read Code |
| 51767 | G666.00 | Pure sensory lacunar syndrome | Read Code |
| 53745 | Gyu6400 | [X]Other cerebral infarction | Read Code |
| 55247 | G65z000 | Impending cerebral ischaemia | Read Code |
| 57315 | G618.00 | Intracerebral haemorrhage, multiple localized | Read Code |
| 62342 | G615.00 | Bulbar haemorrhage | Read Code |
| 90572 | Gyu6500 | [X]Occlusion and stenosis of other precerebral arteries | Read Code |
| 91627 | Gyu6300 | [X]Cerebrl infarctn due / unspcfocclusn or sten/cerebrl artrs | Read Code |
| 92036 | Gyu6600 | [X]Occlusion and stenosis of other cerebral arteries | Read Code |
| 94482 | Gyu6G00 | [X]Cereb infarct due unspocclus/ stenos precerebr arteries | Read Code |
| 96630 | Gyu6F00 | [X]Intracerebral haemorrhage in hemisphere, unspecified | Read Code |
| 101251 | ZV12D00 | [V]Personal history of transient ischaemic attack | Read Code |

**Table S2. Long-term conditions’ definition**

| **Condition** | **Variable Definition** | **Method for Developing Code Lists** | **Physical or Mental Condition** |
| --- | --- | --- | --- |
| Alcohol Problems | Diagnostic code ever recorded | Developed by Cambridge CPRD team based on a list from the Scottish Information Services Division (ISD) [23]. | Mental |
| Anorexia or Bulimia | Diagnostic code ever recorded | Developed by Cambridge CPRD team from searching the CPRD medcode browser. | Mental |
| Asthma (currently treated) | Diagnostic code ever recorded and any asthma prescription in the last 12 months. | Cambridge CPRD team developed the diagnostic code list sourced from QOF [24] and Kontopantelis et al., 2014 [25]. | Physical |
| Atrial Fibrillation | Diagnostic code ever recorded | Diagnostic code list sourced from the QOF [24]. | Physical |
| Blindness and Low Vision | Diagnostic code ever recorded | The Cambridge CPRD team used the Scottish ISD list^4^ to sensitise an iterative search of Readcode lists and terms using the code browser. | Physical |
| Bronchiectasis | Diagnostic code ever recorded | Developed using Scottish ISD list [23] and code choices were confirmed using the CPRD medcode browser search tool. | Physical |
| New diagnosis of Cancer in last five years | Diagnostic code ever recorded in last 5 years | The Cambridge CPRD team developed this using the CPRD medcode browser and codes from Kontopantelis et al., 2014 [25]. | Physical |
| Chronic Kidney Disease | Best of the last 2 GFR readings <60 mL/min* | Our method was a simplified version of the method developed by McDonald et al., 2016 [26]. | Physical |
| Chronic Liver Disease and Viral Hepatitis | Diagnostic code ever recorded | Developed by Cambridge CPRD team based on the list developed by Khan et al., 2010 [27], with chronic hepatitis codes added. | Physical |
| Chronic Obstructive Pulmonary Disease | Diagnostic code ever recorded | Developed by Cambridge CPRD team based on QOF [24]. | Physical |
| Constipation (Treated) | ≥4 laxative prescriptions in last year | Developed by Cambridge CPRD team using the BNF chapter 1.6 [28] and CPRD product code browser to identify laxatives. | Physical |
| Coronary Heart Disease | Diagnostic code ever recorded | Cambridge CPRD team developed this by combining 3 codelists covering coronary revascularisation procedures [25, 29, 30]. The CPRD Read term browser and related hierarchies were searched to add a small number of additional codes based on clinical experience. | Physical |
| Dementia | Diagnostic code ever recorded | Developed based on two published dementia diagnosis lists [27, 31] and was supplemented by a search of the CPRD medical diagnosis browser for additional dementia codes. | Mental |
| Depression or Anxiety | Diagnostic code recorded in last 12 months or ≥4 anti-depressant prescriptions (excluding low dose tricyclics) in last 12 months OR ≥4 anxiolytic/hypnotic prescriptions in the last 12 months | Developed by Cambridge CPRD team based on searching the CPRD medcode browser, BNF chapters 4.1 and 4.3^,^ [28] and CPRD product code browser. | Mental |
| Diabetes | Diagnostic code ever recorded | Developed by Cambridge CPRD team using the CPRD medcode browser and comparing to other pre-existing lists. | Physical |
| Diverticular Disease of Intestine | Diagnostic code ever recorded | Developed by Cambridge CPRD team based on Scottish ISD code list [23]. | Physical |
| Epilepsy (currently treated) | Diagnostic code ever recorded and antiepileptic prescription in last 12 months | Diagnostic codelist was developed by the Cambridge CPRD team and the prescription code list used the drug list from Kontopantelis et al.., 2014 [25]. | Physical |
| Heart Failure | Diagnostic code ever recorded | Developed by the Cambridge CPRD team based on a list from Kontopantelis et al.*.*, 2015 [32]. | Physical |
| Hearing Loss | Diagnostic code ever recorded | Developed by the Cambridge CPRD team using CPRD medcode browser to expand upon the Scottish ISD list [23]. | Physical |
| Hypertension | Diagnostic code ever recorded | This list is from QOF [24]. | Physical |
| Inflammatory Bowel Disease | Diagnostic code ever recorded | Developed by the Cambridge CPRD team based on the Scottish ISD list [23]. | Physical |
| Irritable Bowel Syndrome | Diagnostic code ever recorded or ≥ 4 prescription-only antispasmodic prescription in the last year | An original list developed by the Cambridge CPRD team based on searches of the CPRD medcode browser, BNF chapter 1.2 [28], and CPRD product code browser. | Physical |
| Learning Disability | Diagnostic code ever recorded | Developed by Cambridge CPRD team based on searching the CPRD medcode browser. | Mental |
| Migraine | ≥ 4 prescription-only anti-migraine prescriptions in last year | Developed by the Cambridge CPRD team based on BNF chapter 4.7^.^ [28] and searching CPRD product code browser for triptans. | Physical |
| Multiple Sclerosis | Diagnostic code ever recorded | Developed using Scottish ISD list [23] and code choices were confirmed using the CPRD medcode browser search tool. | Physical |
| Other Psychoactive Substance Misuse | Diagnostic code ever recorded | Developed by the Cambridge CPRD team based on searching the CPRD medcode browser. | Mental |
| Painful Condition | ≥prescription-only analgesics in last 12 months or ≥ 4 specified anti-epileptics in the absence of an epilepsy Read code in last 12 months | Developed by the Cambridge CPRD team based on searching BNF chapter 4.7 [28], and CPRD product code browser. | Physical |
| Parkinson’s Disease | Diagnostic code ever recorded | Developed by the Cambridge CPRD team based on the Scottish ISD list [23]. | Physical |
| Peripheral Vascular Disease | Diagnostic code ever recorded | Developed by Cambridge CPRD team based on searching the CPRD medcode browser. | Physical |
| Prostate Disorders | Diagnostic code ever recorded | Developed by the Cambridge CPRD team based on the Scottish ISD list [23]. | Physical |
| Psoriasis or Eczema | Diagnostic code ever recorded and ≥ 4 related prescriptions in last 12 months (excluding simple emollients) | Diagnostic codes were developed by the Cambridge CPRD team by searching the CPRD medcode browser. Prescription codes were developed by searching BNF chapters 13^.^ [28] and the CPRD product code browser. | Physical |
| Rheumatoid Arthritis, other Inflammatory Polyarthropathies & Systematic connective tissue disorders | Diagnostic code ever recorded | Developed by Cambridge CPRD team based on Scottish ISD code list [23]. | Physical |
| Schizophrenia (and related non-organic psychosis) or bipolar disorder | Diagnostic code ever recorded or Lithium ever prescribed* | Diagnostic codes were developed by the Cambridge CPRD team by searching the CPRD medcode browser. Prescription codes were developed by the Cambridge CPRD team by searching BNF chapters 4.2.1 [28] and the CPRD product code browser. | Mental |
| Chronic Sinusitis | Diagnostic code ever recorded | Developed by Cambridge CPRD team based on searching the CPRD medcode browser. | Physical |
| Stroke & Transient Ischaemic Attack | Diagnostic code ever recorded | Developed by Cambridge CPRD team based on QOF [24]. | Physical |
| Thyroid disorders | Diagnostic code ever recorded | Developed by Cambridge CPRD based on QOF [24]. | Physical |

Note: Further details and the complete code lists are available at https://www.cprdrepos.phpc.cam.ac.uk/cprd-cambridge-codes-lists-gold/
