# Supplementary material for: Clustering of multimorbidity in stroke and transient ischaemic attack survivors: a population-based study
Source: BMC Med. 2026 May 18;24:472. doi: 10.1186/s12916-026-04924-7 (PMC13548555; doi:10.1186/s12916-026-04924-7)
Supplement: Supplementary file 2 [file 12916_2026_4924_MOESM2_ESM.docx]

**Additional file 2**

Correspondence to: Dr Efthalia Massou; [em716@medschl.cam.ac.uk](mailto:em716@medschl.cam.ac.uk).

This addiational file goes along with the main paper and includes results’ details.

Figure S1 presents schematically the overview of patients inclusion, detailing the number of individuals included and excluded in each part of the analysis. IMD scores were available for 16,767 patients, with 52,606 missing values. For 84 of these (0.16%), missing data were replaced using practice-level IMD, resulting in a total of 16,851 patients with available IMD information.

**Figure S1. Flow chart of patients included in each part of the analysis.**

Adults, men and women with stroke/ TIA medical codes in CPRD (database March 2020); N=69,372

Descriptive statistics of LTCs and multimorbidity for the stroke/ TIA survivors’ sample (N=69,372)

Eligible for IMD linkages (N=16,851)

Patient level: N=16,767

Practice level: N=16,851

Patients used for the clustering*:

- 695 patients aged 18-44
- 9,737 patients aged 45-64
- 32,307 patients aged 65-84
- 11,972 patients aged 85+

Patients used for the generalised linear models:

- 556 patients aged 18-44
- 7,789 patients aged 45-64
- 25,845 patients aged 65-84
- 9,577 patients aged 85+

**Note: *** Differences between these numbers and the numbers of multimorbid patients reported in table 1 in the main text, are explained by the exclusion of very rare conditions (i.e., prevalence<1%).

Table S3 presents the demographic characteristics of the overall study population and of patients with multimorbidity.

[Table S3 here]

Table S4 shows morbidity prevalence in the total sample and by age group, with statistically significant differences observed for all LTCs across age groups.

[Table S4 here]

*Latent class analysis*

*Clustering by age strata*

The graphical representations of the key statistics considered in our decision-making process, are given in figures S2a-S2d. Table S5 presents the diagnostic statistics considered when selecting the number of clusters for each age group.

**Figure S2a. Model selection statistics for the age group of 18-44.**

**Figure S2b. Model selection statistics for the age group of 45-64.**

**Figure S2c. Model selection statistics for the age group of 65-84.**

**Figure S2d. Model selection statistics for the age group of 85+.**

[Table S5 here]

The model fit statistics for the age stratum 18-44 indicated that a two-class solution provided the most appropriate balance between model fit and parsimony. The AIC showed an initial decrease up to two classes, after which improvements were minimal, while BIC and adjusted BIC increased steadily with additional classes, suggesting overfitting. Entropy was highest for the two-class model, indicating better classification quality compared to more complex solutions. Taken together, these criteria support the selection of a two-cluster solution as the most interpretable and statistically robust model.

For the age stratum 45-64, we selected the 5-clusters model due to the low BIC SABIC and adjusted-BIC. The 6-clusters model was also considered, however, that model was more complicated in its interpretability due to the overlapping in the leading conditions in two of the six clusters. Given the homogeneity of our population, and the need for interpretable clusters, we selected the 5-clusters model which does not deviate significantly from the competing models in terms of the statistical criteria.

The 3-clusters model was selected for the ages 65-84. The rationale behind this model selection relies on relatively low information criteria and interpretability. Alternative options with more classes, based on lower BIC, SABIC and adjusted BIC criteria, were accompanied by very poor entropy and repetitive leading conditions for the identified clusters.

The 4-clusters model was selected for patients over 85 years. This choice was preferred over the 5-clusters model which had the optimal information criteria values, since the latter would lead to an over-separation highly homogeneous population, without significant gains in clustering quality.

For each of the 14 clusters across the four age strata, we estimated the prevalence of each LTC. The results are given in tables S6-S9 that follow.

[Tables S6, S7, S8, S9 here]

We applied the number of clusters defined using the training sets to the corresponding test sets. The clusters of the training and tests sets and the prevalence of the lead conditions are shown in table S10.

[Table S10 here]

Table S11 presents the bivariate Pearson’s correlation coefficient between the clusters identified in the training and the test set, for each age stratum. The table shows one by one mapping of the clusters identified in the training and the test set with very high Pearson correlations. An exception to this was the clusters of “established CVD” and “CVD risk factors” which were hard to distinguish.

[Table S11 here]

**Multimorbidity clusters and outcomes: age strata 18-44 years old**

Two distinct clusters were identified within the age group of 18-44. A “higher multimorbidity” cluster, characterised by elevated prevalence of depression and painful conditions accounted for 33% of patients. This cluster also showed higher levels of substance misuse and comorbid physical health conditions compared to the rest of the population. Patients in this cluster had substantially higher rates of healthcare utilisation, including GP consultations, prescriptions, and hospital admissions, as well as markedly increased mortality (OR 5.70, 95% CI 4.46–7.51).

In contrast, a “lower morbidity” cluster, accounting for 67% of patients, was characterised by low prevalence across most long-term conditions, with a mean of 1.7 comorbidities per patient. This group had more favourable demographic and clinical profiles, including lower smoking rates, lower BMI, and reduced deprivation levels. Correspondingly, this cluster exhibited the lowest levels of healthcare utilisation and mortality within this age stratum.

**Figure S3. Clustering of multimorbidity in stroke survivors 18-44 years old.**

Notes:

1. The upper panel displays the prevalence of long-term conditions within each latent class, with colour intensity representing prevalence (from low [dark] to high [yellow]). Each column corresponds to a latent class, and each row to a specific condition. The lower panel summarises demographic characteristics and outcomes ( number of consultations in a year, number of new prescriptions in a year, number of hospital admissions in a year, 2-year mortality) across classes. Mortality was analysed using regression models as the primary outcome. Regression analyses for other outcomes are presented in Additional file 2:Table S12.

2. The 2-year mortality logistic regression model was adjusted for gender, deprivation, smoking status, BMI and age.

3. *** *p* < 0.001.

**Generalised linear models**

To assess differences in healthcare utilization and mortality across clusters, we used incidence rate ratios (IRRs) for primary care consultations, new prescriptions, and hospital admissions, and odds ratios (ORs) for two-year mortality. Tables S12-S15 present the associations between multimorbidity clusters, health services utilisation and mortality, when adjusting for gender, deprivation, smoking status, BMI and age.

[Table S12, S13, S14, S15 here]

**Table S3. Demographic characteristics of the study population and multimorbid patients.**

|  | **N (%) the whole sample** | **Multimorbid patients (% of the demographic group)** | **No. of morbidities, median [IQR]** |
| --- | --- | --- | --- |
| **All patients** | 69,372 (100) | 65,687 (94.69) | 3[2-4] |
| **Region** |  |  |  |
| North East | 124 (0.18) | 118 (95.16) | 3 [2,6] |
| North West | 3,639 (5.25) | 3,441 (94.56) | 3 [2,4] |
| Yorkshire and the Humber | 403 (0.58) | 375 (93.05) | 3 [2,4] |
| West Midlands | 3,556 (5.13) | 3,376 (94.94) | 3 [2,4] |
| East of England | 1,180 (1.70) | 1,090 (92.37) | 3 [1,4] |
| South West | 2,082 (3.00) | 1,981 (95.15) | 3 [2,4] |
| South Central | 2,131 (3.07) | 2,016 (94.60) | 3 [2,4] |
| London | 2,775 (4) | 2,601 (93.73) | 3 [2,4] |
| South East Coast | 6,004 (8.65) | 5,622 (93.64) | 3 [2,4] |
| Northern Ireland | 4,645 (6.7) | 4,471 (96.25) | 3 [2,5] |
| Scotland | 25,186 (36.31) | 23,800 (94.50) | 3 [2,4] |
| Wales | 17,647 (25.44) | 16,796 (95.18) | 3 [2,4] |
| **SES*(available only for patients with linkages, N=16,851)** |  |  |  |
| Q1 – least deprived | 2,682(15.92) | 2,505 (93.40) | 3 [2,4] |
| Q2 | 2,035(12.08) | 1,908 (93.76) | 3 [2,4] |
| Q3 | 1,732(10.28) | 1,610 (92.96) | 3 [2,4] |
| Q4 | 1,547(9.18) | 1,460 (94.38) | 3 [2,4] |
| Q5 | 1,998(11.86) | 1,891 (94.64) | 3 [2,4] |
| Q6 | 1,402(8.32) | 1,326 (94.58) | 3 [2,4] |
| Q7 | 1,501(8.91) | 1,427 (95.07) | 3 [2,4] |
| Q8 | 1,364(8.09) | 1,296 (95.01) | 3 [2,4] |
| Q9 | 1,371(8.14) | 1,288 (93.95) | 3 [2,4] |
| Q10 – most deprived | 1,219(7.23) | 1,164 (95.49) | 3 [2,5] |

**Note:** * SES stands for socioeconomic status.

**Table S4. Prevalence of morbidities in the whole sample and by age group.**

| **Morbidity** | **N (%over the whole sample)** | **Age group** | | | |  |
| --- | --- | --- | --- | --- | --- | --- |
|  |  | **18-44** | **45-64** | **65-84** | **85+** | **Pearson chi2 (p-value)** |
|  |  | **N (%)** | **N (%)** | **N (%)** | **N (%)** |  |
| **Stroke & Transient Ischaemic Attack** | 69,372 (100.00) | 1,571 (2.26) | 14,053 (20.26) | 39,831 (57.42) | 13,917 (20.06) |  |
| **Hypertension** | 41,384 (59.65) | 226 (14.39) | 6142(43.71) | 25180(63.22) | 9835(70.67) | 3,734.32 *** |
| **Painful Condition** | 26,666 (38.44) | 299 (19.03) | 4737(33.71) | 15190(38.14) | 6440(46.27) | 745.55 *** |
| **Coronary Heart Disease** | 15,258 (21.99) | 39( 2.48) | 1685(11.99) | 9538(23.95) | 3996(28.71) | 1,622.97 *** |
| **Hearing Loss** | 16,992 (24.49) | 142 (9.04) | 1689(12.02) | 9649(24.22) | 5512(39.61) | 3,105.57 *** |
| **Depression** | 17,890 (25.79) | 392 (24.95) | 4764(33.90) | 9602(24.11) | 3132(22.50) | 621.01 *** |
| **Diabetes** | 15,250 (21.98) | 99( 6.30) | 2732(19.44) | 9704(24.36) | 2715(19.51) | 459.44 *** |
| **Atrial Fibrillation** | 13,084 (18.86) | 37 (2.36) | 840(5.98) | 7886(19.80) | 4321(31.05) | 3,177.57 *** |
| **Chronic Kidney Disease** | 19,429 (28.01) | 57 (3.63) | 1513(10.77) | 11474(28.81) | 6385(45.88) | 4,752.02 *** |
| **Thyroid disorders** | 8,576 (12.36) | 82 (5.22) | 1151(8.19) | 5025(12.62) | 2318(16.66) | 538.91*** |
| **Diverticular Disease of Intestine** | 9,548 (13.76) | 4 (0.25) | 782(5.56) | 5941(14.92) | 2821(20.27) | 1,578.39 *** |
| **Asthma (currently treated)** | 6,881 (9.92) | 167 (10.63) | 1675(11.92) | 4000(10.04) | 1039(7.47) | 158.24 *** |
| **Irritable Bowel Syndrome** | 5,765 (8.31) | 104 (6.62) | 1344(9.56) | 3358(8.43) | 959(6.89) | 72.43 *** |
| **Prostate disorders** | 6,522 (9.40) | 6 (0.38) | 412(2.93) | 4321(10.85) | 1783(12.81) | 1,128.55 *** |
| **Peptic Ulcer Disease** | 4,643 (6.69) | 16 (1.02) | 584(4.16) | 2945(7.39) | 1098(7.89) | 289.11 *** |
| **Anxiety** | 4,956 (7.14) | 102 (6.49) | 984(7.00) | 2656(6.67) | 1214(8.72) | 67.34 *** |
| **COPD** | 7,438 (10.72) | 12 (0.76) | 1134(8.07) | 5087(12.77) | 1204(8.65) | 503.22 *** |
| **Constipation (Treated)** | 9,279 (13.38) | 68 (4.33) | 990(7.04) | 4724(11.86) | 3497(25.13) | 2,334.90 *** |
| **Rheumatoid Arthritis,**  **other Inflammatory Polyarthropathies &**  **Systematic connective tissue disorders** | 4,275 (6.16) | 26 (1.65) | 507(3.61) | 2599(6.53) | 1143(8.21) | 324.05 *** |
| **Alcohol Problems** | 2,616 (3.77) | 62 (3.95) | 1,062 (7.56) | 1,385 (3.48) | 107  (0.77) | 910.40 *** |
| **Peripheral Vascular Disease** | 3,900 (5.62) | 7 (0.45) | 465(3.31) | 2592(6.51) | 836(6.01) | 283.80 *** |
| **New diagnosis of Cancer in last five years** | 3,687 (5.31) | 14 (0.89) | 376(2.68) | 2482(6.23) | 815(5.86) | 330.194 *** |
| **Chronic Sinusitis** | 2,524 (3.64) | 35 (2.23) | 508(3.61) | 1560(3.92) | 421(3.03) | 32.66 *** |
| **Heart Failure** | 4,823 (6.95) | 28 (1.78) | 410(2.92) | 2801(7.03) | 1584(11.38) | 841.05 *** |
| **Blindness and Low Vision** | 2,758 (3.98) | 50 (3.18) | 364(2.59) | 1288(3.23) | 1056(7.59) | 606.35 *** |
| **Dementia** | 5,430 (7.83) | 0 (0.00) | 111(0.79) | 2691(6.76) | 2628(18.88) | 3,519.36*** |
| **Psoriasis or Eczema** | 1,720 (2.48) | 18 (1.15) | 315(2.24) | 1013(2.54) | 374(2.69) | 18.01 *** |
| **Epilepsy (currently treated)** | 2,865 (4.13) | 143 (9.10) | 843(6.00) | 1565(3.93) | 314(2.26) | 349.52 *** |
| **Psychoactive substance misuse (not alcohol)** | 1,103 (1.59) | 97 (6.17) | 513(3.65) | 444(1.11) | 49(0.35) | 786.12 *** |
| **Schizophrenia (and related non-organic psychosis)**  **or bipolar disorder** | 1,149 (1.66) | 32 (2.04) | 318(2.26) | 647(1.62) | 152(1.09) | 60.58 *** |
| **Inflammatory Bowel Disease** | 967 (1.39) | 17 (1.08) | 203(1.44) | 615(1.54) | 132(0.95) | 27.99** |
| **Bronchiectasis** | 1,036 (1.49) | 4 (0.25) | 80(0.57) | 714(1.79) | 238(1.71) | 126.65 *** |
| **Chronic Liver Disease and Viral Hepatitis** | 753 (1.09) | 17 (1.08) | 219(1.56) | 448(1.12) | 69(0.50) | 74.92*** |
| **Parkinson’s Disease** | 803 (1.16) | 0 (0.00) | 30(0.21) | 528(1.33) | 245(1.76) | 181.92 *** |
| **Learning Disability** | 324 (0.47) | 46 (2.93) | 147(1.05) | 118(0.30) | 13(0.09) | 372.81 *** |
| **Migraine** | 200 (0.29) | 9 (0.57) | 101(0.72) | 83(0.21) | 7(0.05) | 131.26 *** |
| **Anorexia** | 258 (0.37) | 7 (0.45) | 78(0.56) | 117(0.29) | 56(0.40) | 19.87 *** |
| **Multiple Sclerosis** | 254 (0.37) | 6 (0.38) | 93(0.66) | 131(0.33) | 24(0.17) | 49.51*** |

**Note:** *** p<0.001; ** p<0.05

**Table S5. Model selection statistics for each age strata.**

| **Age group** | **Classes** | **2** | **3** | **4** | **5** | **6** | **7** | **8** | **9** | **10** |
| --- | --- | --- | --- | --- | --- | --- | --- | --- | --- | --- |
| **18-44** | **Log-Likelihood** | -6254.51 | -6186.81 | -6141.73 | -6094.66 | -6066.72 | -6036.33 | -6011.32 | -5999.38 | -5983.83 |
|  | **AIC** | 12635.02 | 12563.63 | 12537.47 | 12507.32 | 12515.43 | 12518.66 | 12532.63 | 12572.77 | 12605.65 |
|  | **BIC** | 12958.26 | 13051.06 | 13189.09 | 13323.13 | 13495.43 | 13662.85 | 13841.01 | 14045.34 | 14242.41 |
|  | **SABIC** | 12758.15 | 12749.30 | 12785.68 | 12818.08 | 12888.73 | 12954.50 | 13031.01 | 13133.69 | 13229.12 |
|  | **Adjusted BIC** | 12958.37 | 13051.21 | 13189.30 | 13323.39 | 13495.74 | 13663.20 | 13841.42 | 14045.80 | 14242.92 |
|  | **Adjusted AIC** | 12761.02 | 12761.02 | 12761.02 | 12761.02 | 12761.02 | 12761.02 | 12761.02 | 12761.02 | 12761.02 |
|  | **Entropy** | 0.76 | 0.65 | 0.62 | 0.64 | 0.55 | 0.56 | 0.53 | 0.36 | 0.28 |
|  | **Likelihood Ratio** | 2468.68 | 2333.29 | 2243.13 | 2148.99 | 2093.10 | 2032.32 | 1982.29 | 1958.43 | 1927.32 |
|  | **Class Sizes (%)** | 33.15, 66.85 | 64.03, 13.11, 22.86 | 3.26, 54.89, 18.51, 23.34 | 4.98, 2.52, 19.52, 10.67, 62.32 | 14.93, 10.11, 4.04, 7.93, 3.18, 59.82 | 5.08, 1.31, 57.84, 15.69, 3.69, 3.09, 13.3 | 1.6, 53.29, 5.39, 3.7, 3.36, 14.82, 1.1, 16.75 | 3.7, 4.25, 14.75, 21.08, 1.22, 2.1, 0.61, 43.25, 9.03 | 10.31, 4.61, 13.47, 4.24, 0.24, 5.2, 22.79, 34.45, 2.34, 2.33 |
| **45-64** | **Log-Likelihood** | -86,423.80 | -85,875.20 | -85,462.13 | -85,254.61 | -85,080.14 | -84,951.41 | -84,858.47 | -84,776.03 | -84,693.04 |
|  | **AIC** | 172,981.59 | 171,952.40 | 171,194.26 | 170,847.22 | 170,566.28 | 170,376.81 | 170,258.95 | 170,162.06 | 170,064.08 |
|  | **BIC** | 173,472.07 | 172,691.78 | 172,182.53 | 172,084.39 | 172,052.35 | 172,111.78 | 172,242.81 | 172,394.82 | 172,545.74 |
|  | **SABIC** | 173,259.15 | 172,370.81 | 171,753.52 | 171,547.33 | 171,407.24 | 171,358.63 | 171,381.61 | 171,425.57 | 171,468.45 |
|  | **Adjusted BIC** | 173,472.08 | 172,691.80 | 172,182.56 | 172,084.42 | 172,052.38 | 172,111.82 | 172,242.86 | 172,394.88 | 172,545.81 |
|  | **Adjusted AIC** | 173,115.59 | 172,154.40 | 171,464.26 | 171,185.22 | 170,972.28 | 170,850.81 | 170,800.95 | 170,772.06 | 170,742.08 |
|  | **Entropy** | 0.74 | 0.59 | 0.54 | 0.45 | 0.31 | 0.23 | 0.21 | 0.17 | 0.12 |
|  | **Likelihood Ratio** | 28,628.74 | 27,531.55 | 26,705.41 | 26,290.37 | 25,941.43 | 25,683.96 | 25,498.09 | 25,333.21 | 25,167.23 |
|  | **Class Sizes (%)** | 61.09, 38.91 | 15.08, 28.87, 56.06 | 14.48, 24.09, 55.25, 6.18 | 15.11, 49.15, 4.94, 6.08, 24.72 | 6.96, 8.75, 23.55, 14.24, 5.21, 41.3 | 17.04, 6.35, 40.98, 8.31, 8.54, 4.78, 14.02 | 7.57, 14.09, 7.03, 6.09, 7.81, 42.6, 4.52, 10.29 | 7.95, 14.47, 4.33, 7.25, 10.17, 5.65, 5.4, 3.67, 41.11 | 2.75, 15.16, 9.86, 2.86, 6.56, 39.08, 5.39, 4.24, 8.39, 5.71 |
|  | **Classes** | **2** | **3** | **4** | **5** | **6** | **7** | **8** | **9** | **10** |
| **65-84** | **Log-Likelihood** | -314,129.59 | -312,191.07 | -311,482.80 | -310,877.06 | -310,389.93 | -309,966.61 | -309,615.67 | -309,365.58 | -309,166.10 |
|  | **AIC** | 628,393.18 | 624,584.14 | 623,235.60 | 622,092.12 | 621,185.86 | 620,407.23 | 619,773.34 | 619,341.17 | 619,010.20 |
|  | **BIC** | 628,953.82 | 625,429.29 | 624,365.25 | 623,506.27 | 622,884.51 | 622,390.39 | 622,041.01 | 621,893.33 | 621,846.87 |
|  | **SABIC** | 628,740.90 | 625,108.31 | 623,936.22 | 622,969.19 | 622,239.38 | 621,637.20 | 621,179.77 | 620,924.05 | 620,769.53 |
|  | **Adjusted BIC** | 628,953.83 | 625,429.29 | 624,365.26 | 623,506.28 | 622,884.53 | 622,390.40 | 622,041.02 | 621,893.35 | 621,846.89 |
|  | **Adjusted AIC** | 628,527.18 | 624,786.14 | 623,505.60 | 622,430.12 | 621,591.86 | 620,881.23 | 620,315.34 | 619,951.17 | 619,688.20 |
|  | **Entropy** | 0.66 | 0.51 | 0.40 | 0.40 | 0.21 | 0.11 | 0.11 | 0.05 | 0.02 |
|  | **Likelihood Ratio** | 104,250.26 | 100,373.23 | 98,956.68 | 97,745.20 | 96,770.94 | 95,924.31 | 95,222.43 | 94,722.25 | 94,323.28 |
|  | **Class Sizes (%)** | 41.06, 58.94 | 20.56, 25.17, 54.27 | 19.46, 11.32, 17.57, 51.65 | 19.14, 17.25, 2.29, 9.83, 51.5 | 10.76, 2.18, 20.04, 38.77, 12.6, 15.65 | 34.5, 14.25, 12.13, 7.2, 18.05, 11.79, 2.07 | 5.24, 6.32, 13.04, 34.96, 18.06, 9.33, 11.24, 1.8 | 6.34, 5.03, 18.13, 33.22, 9.95, 1.78, 6.61, 12.93, 6 | 1.76, 17.01, 12.76, 5.17, 5.5, 6.97, 4.96, 32.49, 7.05, 6.34 |
|  | **Classes** | **2** | **3** | **4** | **5** | **6** | **7** | **8** | **9** | **10** |
| **85+** | **Log-Likelihood** | -118,701.97 | -117,999.19 | -117,703.90 | -117,534.23 | -117,402.44 | -117,278.43 | -117,160.40 | -117,081.58 | -117,009.46 |
|  | **AIC** | 237,537.95 | 236,200.38 | 235,677.80 | 235,406.46 | 235,210.88 | 235,030.85 | 234,862.80 | 234,773.17 | 234,696.93 |
|  | **BIC** | 238,028.22 | 236,939.44 | 236,665.66 | 236,643.12 | 236,696.33 | 236,765.10 | 236,845.84 | 237,005.00 | 237,177.55 |
|  | **SABIC** | 237,815.30 | 236,618.48 | 236,236.64 | 236,106.06 | 236,051.22 | 236,011.94 | 235,984.63 | 236,035.75 | 236,100.26 |
|  | **Adjusted BIC** | 238,028.23 | 236,939.46 | 236,665.68 | 236,643.15 | 236,696.37 | 236,765.14 | 236,845.88 | 237,005.05 | 237,177.62 |
|  | **Adjusted AIC** | 237,671.95 | 236,402.38 | 235,947.80 | 235,744.46 | 235,616.88 | 235,504.85 | 235,404.80 | 235,383.17 | 235,374.93 |
|  | **Entropy** | 0.64 | 0.45 | 0.31 | 0.19 | 0.10 | 0.00 | -0.03 | -0.11 | -0.15 |
|  | **Likelihood Ratio** | 51,821.93 | 50,416.36 | 49,825.78 | 49,486.44 | 49,222.86 | 48,974.84 | 48,738.78 | 48,581.15 | 48,436.91 |
|  | **Class Sizes (%)** | 39.51, 60.49 | 30.13, 46.9, 22.97 | 44.14, 19.24, 18.94, 17.68 | 18.21, 11.3, 15.24, 16, 39.26 | 17.02, 16.27, 15.26, 3.35, 28.65, 19.45 | 20.5, 15.46, 20.51, 15.37, 10.96, 3.23, 13.97 | 19.19, 15.7, 11.43, 24.41, 8.57, 9, 2.84, 8.86 | 8.22, 6.17, 13.55, 9.58, 19.8, 8.52, 21.55, 3.15, 9.47 | 3.38, 7.09, 8.87, 21.38, 11.02, 6.98, 18.52, 7.11, 6.58, 9.09 |

**Table S6. Prevalence of each LTC in the clusters of multimorbidity among patients aged 18-44.**

| **Long-term condition (LTC)** | **Lower morbidity** | **Higher multimorbidity** |
| --- | --- | --- |
| **Alcohol** | 0.018 | 0.090 |
| **Diverticular Disease of Intestine** | 0.001 | 0.000 |
| **Anxiety** | 0.006 | 0.196 |
| **Asthma** | 0.073 | 0.170 |
| **Atrial Fibrillation** | 0.019 | 0.028 |
| **Blindness and Low Vision** | 0.025 | 0.051 |
| **Bronchiectasis** | 0.001 | 0.008 |
| **COPD** | 0.000 | 0.024 |
| **Cancer** | 0.008 | 0.008 |
| **CKD** | 0.013 | 0.088 |
| **Chronic Liver Disease** | 0.000 | 0.031 |
| **Chronic Sinusitis** | 0.019 | 0.003 |
| **Constipation** | 0.006 | 0.111 |
| **Coronary Heart Disease** | 0.014 | 0.047 |
| **Dementia** | 0.000 | 0.000 |
| **Depression** | 0.072 | 0.591 |
| **Diabetes** | 0.003 | 0.122 |
| **Epilepsy** | 0.007 | 0.133 |
| **Hearing Loss** | 0.074 | 0.118 |
| **Heart Failure** | 0.006 | 0.041 |
| **Hypertension** | 0.100 | 0.249 |
| **Inflammatory Bowel Disease** | 0.007 | 0.019 |
| **Irritable Bowel Syndrome** | 0.028 | 0.139 |
| **Painful Condition** | 0.032 | 0.525 |
| **Parkinson’s Disease** | 0.000 | 0.000 |
| **Peptic Ulcer Disease** | 0.000 | 0.024 |
| **Peripheral Vascular Disease** | 0.001 | 0.012 |
| **Prostate disorders** | 0.002 | 0.010 |
| **Psoriasis or Eczema** | 0.005 | 0.021 |
| **Psychoactive Substance Misuse** | 0.029 | 0.126 |
| **Psychosis** | 0.004 | 0.052 |
| **Rheumatoid Arthritis** | 0.010 | 0.024 |
| **Thyroid disorders** | 0.035 | 0.086 |

**Table S7. Prevalence of each LTC in the clusters of multimorbidity among patients aged 45-64.**

| **Long-term condition (LTC)** | **Alcocol and substance misuse** | **Established**  **CVD** | **Poor mental health** | **Lower morbidity better outcomes** | **CVD risk factors** |
| --- | --- | --- | --- | --- | --- |
| **Alcohol** | 0.714 | 0.080 | 0.053 | 0.022 | 0.024 |
| **Diverticular Disease of Intestine** | 0.042 | 0.059 | 0.105 | 0.031 | 0.053 |
| **Anxiety** | 0.145 | 0.070 | 0.208 | 0.010 | 0.011 |
| **Asthma** | 0.134 | 0.208 | 0.248 | 0.075 | 0.038 |
| **Atrial Fibrillation** | 0.033 | 0.298 | 0.035 | 0.051 | 0.061 |
| **Blindness and Low Vision** | 0.013 | 0.071 | 0.045 | 0.014 | 0.028 |
| **Bronchiectasis** | 0.010 | 0.023 | 0.011 | 0.003 | 0.000 |
| **COPD** | 0.214 | 0.223 | 0.148 | 0.035 | 0.014 |
| **Cancer** | 0.034 | 0.033 | 0.036 | 0.017 | 0.043 |
| **CKD** | 0.062 | 0.358 | 0.133 | 0.037 | 0.238 |
| **Chronic Liver Disease** | 0.096 | 0.031 | 0.014 | 0.006 | 0.010 |
| **Chronic Sinusitis** | 0.021 | 0.026 | 0.059 | 0.033 | 0.019 |
| **Constipation** | 0.098 | 0.133 | 0.205 | 0.007 | 0.022 |
| **Coronary Heart Disease** | 0.140 | 0.637 | 0.128 | 0.048 | 0.166 |
| **Dementia** | 0.013 | 0.006 | 0.018 | 0.001 | 0.009 |
| **Depression** | 0.524 | 0.432 | 0.763 | 0.129 | 0.234 |
| **Diabetes** | 0.120 | 0.535 | 0.248 | 0.055 | 0.487 |
| **Epilepsy** | 0.088 | 0.062 | 0.112 | 0.037 | 0.033 |
| **Hearing Loss** | 0.105 | 0.141 | 0.169 | 0.106 | 0.096 |
| **Heart Failure** | 0.014 | 0.408 | 0.006 | 0.008 | 0.014 |
| **Hypertension** | 0.375 | 0.582 | 0.433 | 0.309 | 0.837 |
| **Inflammatory Bowel Disease** | 0.005 | 0.004 | 0.024 | 0.012 | 0.012 |
| **Irritable Bowel Syndrome** | 0.044 | 0.087 | 0.213 | 0.062 | 0.042 |
| **Painful Condition** | 0.501 | 0.548 | 0.750 | 0.106 | 0.291 |
| **Parkinson’s Disease** | 0.003 | 0.002 | 0.004 | 0.002 | 0.000 |
| **Peptic Ulcer Disease** | 0.122 | 0.087 | 0.057 | 0.022 | 0.035 |
| **Peripheral Vascular Disease** | 0.087 | 0.180 | 0.028 | 0.006 | 0.062 |
| **Prostate disorders** | 0.041 | 0.042 | 0.033 | 0.024 | 0.037 |
| **Psoriasis or Eczema** | 0.419 | 0.016 | 0.033 | 0.001 | 0.008 |
| **Psychoactive Substance Misuse** | 0.019 | 0.029 | 0.040 | 0.013 | 0.020 |
| **Psychosis** | 0.068 | 0.013 | 0.052 | 0.006 | 0.004 |
| **Rheumatoid Arthritis** | 0.004 | 0.063 | 0.075 | 0.021 | 0.027 |
| **Thyroid disorders** | 0.021 | 0.077 | 0.153 | 0.058 | 0.069 |

**Table S8. Prevalence of each LTC in the clusters of multimorbidity among patients aged 65-84.**

| **Long-term condition (LTC)** | **Poor mental health** | **Established CVD** | **Lower morbidity better outcomes** |
| --- | --- | --- | --- |
| **Alcohol** | 0.081 | 0.018 | 0.020 |
| **Diverticular Disease of Intestine** | 0.227 | 0.189 | 0.096 |
| **Anxiety** | 0.204 | 0.049 | 0.009 |
| **Asthma** | 0.180 | 0.117 | 0.058 |
| **Atrial Fibrillation** | 0.129 | 0.414 | 0.148 |
| **Blindness and Low Vision** | 0.045 | 0.049 | 0.021 |
| **Bronchiectasis** | 0.033 | 0.020 | 0.010 |
| **COPD** | 0.218 | 0.173 | 0.067 |
| **Cancer** | 0.068 | 0.082 | 0.054 |
| **CKD** | 0.262 | 0.559 | 0.196 |
| **Chronic Liver Disease** | 0.018 | 0.017 | 0.005 |
| **Chronic Sinusitis** | 0.059 | 0.033 | 0.033 |
| **Constipation** | 0.300 | 0.114 | 0.035 |
| **Coronary Heart Disease** | 0.249 | 0.565 | 0.108 |
| **Dementia** | 0.151 | 0.057 | 0.032 |
| **Depression** | 0.594 | 0.192 | 0.095 |
| **Diabetes** | 0.247 | 0.459 | 0.160 |
| **Epilepsy** | 0.070 | 0.023 | 0.032 |
| **Hearing Loss** | 0.269 | 0.292 | 0.211 |
| **Heart Failure** | 0.039 | 0.273 | 0.007 |
| **Hypertension** | 0.620 | 0.767 | 0.590 |
| **Inflammatory Bowel Disease** | 0.022 | 0.016 | 0.012 |
| **Irritable Bowel Syndrome** | 0.166 | 0.069 | 0.049 |
| **Painful Condition** | 0.702 | 0.448 | 0.205 |
| **Parkinson’s Disease** | 0.027 | 0.010 | 0.008 |
| **Peptic Ulcer Disease** | 0.109 | 0.104 | 0.046 |
| **Peripheral Vascular Disease** | 0.074 | 0.152 | 0.029 |
| **Prostate disorders** | 0.096 | 0.158 | 0.096 |
| **Psoriasis or Eczema** | 0.038 | 0.003 | 0.003 |
| **Psychoactive Substance Misuse** | 0.036 | 0.035 | 0.017 |
| **Psychosis** | 0.045 | 0.006 | 0.006 |
| **Rheumatoid Arthritis** | 0.098 | 0.083 | 0.042 |
| **Thyroid disorders** | 0.173 | 0.149 | 0.096 |

**Table S9. Prevalence of each LTC in the clusters of multimorbidity among patients aged 85+.**

| **Long-term condition (LTC)** | **Lower morbidity better outcomes** | **Poor mental health** | **Established CVD** | **Dementia-dominant** |
| --- | --- | --- | --- | --- |
| **Alcohol** | 0.006 | 0.014 | 0.007 | 0.004 |
| **Diverticular Disease of Intestine** | 0.140 | 0.449 | 0.201 | 0.142 |
| **Anxiety** | 0.020 | 0.170 | 0.049 | 0.203 |
| **Asthma** | 0.051 | 0.207 | 0.055 | 0.023 |
| **Atrial Fibrillation** | 0.225 | 0.274 | 0.582 | 0.267 |
| **Blindness and Low Vision** | 0.055 | 0.089 | 0.091 | 0.092 |
| **Bronchiectasis** | 0.010 | 0.054 | 0.009 | 0.000 |
| **COPD** | 0.056 | 0.202 | 0.099 | 0.028 |
| **Cancer** | 0.063 | 0.086 | 0.053 | 0.035 |
| **CKD** | 0.391 | 0.415 | 0.725 | 0.384 |
| **Chronic Liver Disease** | 0.158 | 0.397 | 0.568 | 0.205 |
| **Chronic Sinusitis** | 0.023 | 0.082 | 0.019 | 0.013 |
| **Constipation** | 0.080 | 0.441 | 0.162 | 0.546 |
| **Coronary Heart Disease** | 0.003 | 0.003 | 0.011 | 0.004 |
| **Dementia** | 0.125 | 0.165 | 0.107 | 0.453 |
| **Depression** | 0.071 | 0.389 | 0.136 | 0.516 |
| **Diabetes** | 0.155 | 0.197 | 0.333 | 0.148 |
| **Epilepsy** | 0.014 | 0.024 | 0.021 | 0.040 |
| **Hearing Loss** | 0.371 | 0.533 | 0.402 | 0.331 |
| **Heart Failure** | 0.015 | 0.112 | 0.401 | 0.062 |
| **Hypertension** | 0.675 | 0.714 | 0.816 | 0.672 |
| **Inflammatory Bowel Disease** | 0.007 | 0.022 | 0.005 | 0.006 |
| **Irritable Bowel Syndrome** | 0.031 | 0.228 | 0.032 | 0.050 |
| **Painful Condition** | 0.257 | 0.723 | 0.439 | 0.714 |
| **Parkinson’s Disease** | 0.010 | 0.028 | 0.008 | 0.030 |
| **Peptic Ulcer Disease** | 0.058 | 0.139 | 0.096 | 0.047 |
| **Peripheral Vascular Disease** | 0.029 | 0.095 | 0.123 | 0.029 |
| **Prostate disorders** | 0.127 | 0.181 | 0.194 | 0.027 |
| **Psoriasis or Eczema** | 0.019 | 0.055 | 0.022 | 0.023 |
| **Psychoactive Substance Misuse** | 0.001 | 0.012 | 0.001 | 0.001 |
| **Psychosis** | 0.007 | 0.020 | 0.002 | 0.025 |
| **Rheumatoid Arthritis** | 0.065 | 0.134 | 0.084 | 0.078 |
| **Thyroid disorders** | 0.130 | 0.187 | 0.205 | 0.203 |

**Table S10. Cluster solutions across age strata.**

| **Age strata** | **Label & class size (%)** | **Top 3 most prevalent morbidities (prevalence)** | **Class size (%)** | **Top 3 most prevalent morbidities (prevalence)** |
| --- | --- | --- | --- | --- |
|  | **Training set** | | **Test set** | |
| **18-44** | **Lower multimorbidity (67%)** | Hypertension (0.10) | **73%** | Depression (0.13) |
|  |  | Hearing Loss (0.07) |  | Hypertension (0.11) |
|  |  | Asthma (0.07) |  | Hearing Loss (0.09) |
|  | **Higher multimorbidity (33%)** | Depression (0.59) | **27%** | Depression (0.65) |
|  |  | Painful Condition (0.53) |  | Painful Condition (0.64) |
|  |  | Hypertension (0.25) |  | Hypertension (0.14) |
| **45-64** | **Alcohol and substance misuse (6%)** | Alcohol (0.71) | **7%** | Alcohol (0.66) |
|  |  | Depression (0.52) |  | Depression (0.53) |
|  |  | Painful Condition (0.50) |  | PSM (0.40) |
|  | **Established CVD (5%)** | Coronary Heart Disease (0.64) | **6%** | Painful Condition (0.83) |
|  |  | Hypertension (0.58) |  | Depression (0.72) |
|  |  | Diabetes (0.54) |  | Hypertension (0.57) |
|  | **Poor mental health (25%)** | Depression (0.76) | **21%** | Painful Condition (0.76) |
|  |  | Painful Condition (0.75) |  | Depression (0.68) |
|  |  | Hypertension (0.43) |  | Hypertension (0.36) |
|  | **Lower morbidity better outcomes (49%)** | Hypertension (0.31) | **50%** | Hypertension (0.36) |
|  |  | Depression (0.13) |  | Depression (0.12) |
|  |  | Diabetes (0.12) |  | Hearing Loss (0.10) |
|  | **CVD risk factors (15%)** | Hypertension (0.84) | **16%** | Hypertension (0.75) |
|  |  | Diabetes (0.49) |  | Diabetes (0.53) |
|  |  | Painful Condition (0.29) |  | Painful Condition (0.37) |
| **65-84** | **Poor mental health (25%)** | Painful Condition (0.76) | **28%** | Painful Condition (0.72) |
|  |  | Hypertension (0.62) |  | Hypertension (0.63) |
|  |  | Depression (0.59) |  | Depression (0.58) |
|  | **Established CVD (21%)** | Hypertension (0.77) | **19%** | Hypertension (0.74) |
|  |  | Coronary Heart Disease (0.57) |  | Coronary Heart Disease (0.63) |
|  |  | Chronic Kidney Disease (0.57) |  | Chronic Kidney Disease (0.60) |
|  | **Lower morbidity better outcomes (54%)** | Hypertension (0.59) | **53%** | Hypertension (0.58) |
|  |  | Hearing Loss (0.21) |  | Hearing Loss (0.22) |
|  |  | Painful Condition (0.21) |  | Coronary Kidney Disease (0.19) |
| **85+** | **Lower morbidity better outcomes**  **(44%)** | Hypertension (0.68) | **41%** | Hypertension (0.71) |
|  |  | Chronic Kidney Disease (0.39) |  | Chronic Kidney Disease (0.40) |
|  |  | Hearing Loss (0.37) |  | Hearing Loss (0.35) |
|  | **Poor mental health (19%)** | Painful Condition (0.72) | **18%** | Hypertension (0.69) |
|  |  | Hypertension (0.71) |  | Painful condition (0.64) |
|  |  | Hearing Loss (0.53) |  | Hearing Loss (0.57) |
|  | **Established CVD (18%)** | Hypertension (0.81) | **17%** | Hypertension (0.83) |
|  |  | Chronic Kidney Disease (0.73) |  | Chronic Kidney Disease (0.78) |
|  |  | Coronary Heart Disease (0.73) |  | Atrial Fibrillation (0.64) |
|  | **Dementia-dominant (19%)** | Painful Condition (0.71) | **24%** | Painful Condition (0.69) |
|  |  | Hypertension (0.67) |  | Hypertension (0.60) |
|  |  | Constipation (0.55) |  | Constipation (0.59) |

**Table S11. Pearson’s correlation coefficient between the training and the test clusters.**

| **Age group** |  | **Clusters of multimorbidity in the training set (Top 3 most prevalent morbidities)** | | | | |
| --- | --- | --- | --- | --- | --- | --- |
| **18-44** | **Clusters of multimorbidity in the test set** | **Lower multimorbidity (hypertension, hearing loss, asthma)** | **Higher multimorbidity (Deperession, painful condition, hypertension)** |  |  |  |
|  | **Depression, hypertension, hearing loss** | **0.927** | 0.402 |  |  |  |
|  | **Depression, painful condition, hypertension** | 0.540 | **0.971** |  |  |  |
| **45-64** |  | **Alcohol and substance misuse (alcohol, depression, painful condition)** | **Established CVD (Coronary heart disease, hypertension, diabetes)** | **Poor mental health (Depression, painful condition, hypertension)** | **Lower morbidity better outcomes (hypertension, depression, diabetes)** | **CVD risk factors (hypertension, diabetes, painful condition)** |
|  | **Painful condition, Depression, Hypertension** | 0.603 | 0.552 | **0.984** | 0.642 | 0.523 |
|  | **Painful condition, Depression, Hypertension** | 0.514 | **0.790** | **0.928** | 0.656 | 0.643 |
|  | **Hypertension, Diabetes, Painful condition** | 0.410 | **0.855** | 0.660 | 0.817 | **0.976** |
|  | **Hypertension, Depression, Hearing loss** | 0.370 | 0.610 | 0.561 | **0.981** | 0.883 |
|  | **Alcohol, Depression, PSM** | **0.981** | 0.322 | 0.560 | 0.403 | 0.356 |
| **65-84** |  | **Poor mental health (painful condition, hypertension, depression)** | **Established CVD (Hypertension, coronary heart disease, chronic kidney disease)** | **Lower morbidity better outcomes (Hypertension, hearing loss, painful condition)** |  |  |
|  | **Hypertension, Hearing loss, Chronic kidney disease** | 0.702 | 0.850 | **0.998** |  |  |
|  | **Hypertension, Coronary heart disease, Chronic kidney disease** | 0.656 | **0.996** | 0.806 |  |  |
|  | **Painful condition, Hypertension, Depression,** | **0.999** | 0.670 | 0.722 |  |  |
| **85+** |  | **Lower morbidity better outcomes (Hypertension, chronic kidney disease, hearing loss)** | **Poor mental health (Painful condition, hypertension, hearing loss)** | **Established CVD (Hypertension, chronic hidney disease, coronary heart disease)** | **Dementia-dominant (Painful condition, hypertension, constipation)** |  |
|  | **Hypertension, Chronic kidney disease, Atrial fibrillation** | 0.863 | 0.773 | **0.981** | 0.737 |  |
|  | **Hypertension, Chronic Kidney disease, Hearing loss** | **0.995** | 0.782 | 0.883 | 0.689 |  |
|  | **Hypertension, Painful condition, Hearing loss** | 0.854 | **0.959** | 0.853 | 0.750 |  |
|  | **Painful condition, Hypertension, Constipation** | 0.666 | 0.870 | 0.569 | **0.989** |  |

**Table S12. Association between clusters of multimorbidity, health services utilisation and mortality, for the 18-44 age group.**

|  | **1-year consultations** | **1-year new prescriptions** | **1-year hospital admissions** | **2-year mortality** |
| --- | --- | --- | --- | --- |
|  | **IRR (95% CI)** | **IRR (95% CI)** | **IRR (95% CI)** | **OR (95% CI)** |
| **Cluster** |  |  |  |  |
| **Lower multimorbidity (Hypertension, hearing loss, asthma)** | 1 | 1 | 1 | 1 |
| **Higher multimorbidity (Deperession, painful condition, hypertension)** | 2.26(1.75-2.91)*** | 1.65(1.18-2.29)** | 1.39(0.83-2.33) | 5.70(0.46-70.51) |
| **Gender** |  |  |  |  |
| **Male** | 1 | 1 | 1 | 1 |
| **Female** | 1.16(0.92-1.47) | 1.60(1.16-2.20)** | 1.49(0.88-2.53) | 1.28(0.11-15.04) |
| **SES** |  |  |  |  |
| **Q1 – least deprived** | 1 | 1 | 1 | Socioeconomic status (SES) was not included in this model due to complete separation, as no events were observed across SES categories. |
| **Q2** | 0.84(0.50-1.39) | 0.50(0.24-1.04)* | 0.46(1.10-2.03) |  |
| **Q3** | 0.78(0.48-1.29) | 0.62(0.31-1.22) | 1.23(0.35-4.30) |  |
| **Q4** | 1.10(0.66- 1.85) | 1.02(0.50-2.10) | 0.43(0.69-2.73) |  |
| **Q5** | 1.08(0.69-1.70) | 0.53(0.28-0.98) | 0.82(0.29-2.31) |  |
| **Q6** | 1.14(0.71-1.84) | 0.67(0.34-1.33) | 1.56(0.61-4.01) |  |
| **Q7** | 0.85(0.55-1.31) | 0.62(0.34-1.14) | 0.52(0.17-1.58) |  |
| **Q8** | 0.76(0.48-1.23) | 0.52(0.26-1.03) | 0.99(0.38-2.61) |  |
| **Q9** | 0.92(0.58-1.45) | 0.73(0.40-1.33) | 0.80(0.30-2.19) |  |
| **Q10 – most deprived** | 0.90(0.55-1.47) | 1.10(0.57-2.12) | 0.85(0.29-2.48) |  |
| **Smoking status** |  |  |  |  |
| **Non-smoker** | 1 | 1 | 1 | 1 |
| **Ex-smoker** | 0.99(0.74-1.33) | 0.95(0.63-1.44) | 1.37(0.69-2.74) | 1.18(0.10-13.49) |
| **Current smoker** | 1.10(0.82-1.48) | 1.15(0.78-1.69) | 1.58(0.80-3.09) | This category was omitted from the model due to insufficient data (no events) |
| **BMI** | 1.01(1.00-1.03) | 1.01(0.99-1.03) | 1.00(0.97-1.02) | 1.01(0.89-1.15) |
| **Age** | 1.00(0.98-1.02) | 1.00(0.97-1.03) | 0.99(0.94-1.04) | 0.95(0.78-1.16) |

Note:* p<0.1   ** p<0.05   *** p<0.01

**Table S13. Association between clusters of multimorbidity, health services utilisation and mortality, for the 45-64 age group.**

|  | **1-year consultations** | **1-year new prescriptions** | **1-year hospital admissions** | **2-year mortality** |
| --- | --- | --- | --- | --- |
|  | **IRR (95% CI)** | **IRR (95% CI)** | **IRR (95% CI)** | **OR (95% CI)** |
| **Cluster** |  |  |  |  |
| **Alcohol and substance misuse (alcohol, depression, painful condition)** | 1.91(1.53-2.38)** | 2.01(1.54-2.64)*** | 0.63(0.40-0.98)* | 1.03(0.13-8.27) |
| **Established CVD (Coronary heart disease, hypertension, diabetes)** | 2.56(2.11-3.1)*** | 1.92(1.53-2.41)*** | 2.33(1.72-3.15)*** | 9.75(3.75-25.32)*** |
| **Poor mental health (Depression, painful condition, hypertension)** | 1.96(1.78-2.17)*** | 2.29(2.04-2.59)*** | 1.61(1.32-1.95)*** | 3.27(1.52-7.04)** |
| **Lower morbidity better outcomes (hypertension, depression, diabetes)** | 1 | 1 | 1 | 1 |
| **CVD risk factors (hypertension, diabetes, painful condition)** | 1.67(1.46-1.91)*** | 1.43(1.21-1.70)*** | 0.69(0.51-0.93)* | 1.65(0.51-5.31)* |
| **Gender** |  |  |  |  |
| **Male** | 1 | 1 | 1 | 1 |
| **Female** | 1.12(1.04-1.22)** | 1.25(1.13-1.39)*** | 1.10(0.94-1.30) | 1.20(0.63-2.28) |
| **SES** |  |  |  |  |
| **Q1 – least deprived** | 1 | 1 | 1 | 1 |
| **Q2** | 1.08(0.92-1.28) | 1.38(1.11-1.72)** | 0.84(0.56-1.26) | 0.32(0.03-2.92) |
| **Q3** | 1.26(1.07-1.49)** | 1.13(0.9-1.4) | 1.36(0.96-1.94) | 1.45(0.35-6.03) |
| **Q4** | 1.28(1.08-1.51)** | 1.52(1.22-1.89)*** | 1.31(0.92-1.88) | 0.75(0.13-4.2) |
| **Q5** | 1.22(1.04-1.43)* | 1.56(1.27-1.91)*** | 0.83(0.58-1.18) | 0.93(0.2-4.32) |
| **Q6** | 1.41(1.19-1.67)*** | 1.50(1.21-1.87)*** | 0.92(0.64-1.33) | 0.98(0.21-4.58) |
| **Q7** | 1.33(1.12-1.57)*** | 1.77(1.43-2.2)*** | 1.51(1.07-2.11)* | 1.49(0.38-5.81) |
| **Q8** | 1.20(1.02-1.42)* | 1.46(1.18-1.81)*** | 0.97(0.68-1.39) | 2.08(0.58-7.42) |
| **Q9** | 1.21(1.04-1.41)** | 1.44(1.18-1.75)*** | 1.33(0.96-1.83) | 1.11(0.3-4.13) |
| **Q10 – most deprived** | 1.28(1.09-1.51)** | 1.43(1.16-1.77)** | 1.48(1.06-2.06)* | 1.82(0.51-6.49) |
| **Smoking status** |  |  |  |  |
| **Non-smoker** | 1 | 1 | 1 | 1 |
| **Ex-smoker** | 1.06(0.97-1.16) | 1.07(0.96-1.21) | 0.87(0.72-1.04) | 0.74(0.33-1.63) |
| **Current smoker** | 1.10(0.98-1.22) | 1.29(1.13-1.46)*** | 0.96(0.78-1.18) | 1.69(0.80-3.61) |
| **BMI** | 1.01(1-1.02)** | 1.02(1.01-1.03)*** | 0.98(0.96-0.99)*** | 1.00(0.95-1.05) |
| **Age** | 1.00(0.99-1.01) | 1.01(1-1.02) | 0.99(0.97-1.00) | 1.06(0.99-1.13) |

Note:* p<0.1   ** p<0.05   *** p<0.01

**Table S14. Association between clusters of multimorbidity, health services utilisation and mortality, for the 65-84 age group.**

|  | **1-year consultations** | **1-year new prescriptions** | **1-year hospital admissions** | **2-year mortality** |
| --- | --- | --- | --- | --- |
|  | **IRR (95% CI)** | **IRR (95% CI)** | **IRR (95% CI)** | **OR (95% CI)** |
| **Clusters** |  |  |  |  |
| **Poor mental health (painful condition, hypertension, depression)** | 1.56(1.47-1.65)*** | 1.47(1.37-1.58)*** | 1.56(1.41-1.72)*** | 2.37(1.84-3.05)*** |
| **Established CVD (Hypertension, coronary heart disease, chronic kidney disease)** | 1.49(1.41-1.58)*** | 1.68(1.57-1.80)*** | 1.35(1.23-1.48)*** | 2.47(1.93-3.17)*** |
| **Lower morbidity better outcomes (Hypertension, hearing loss, painful condition)** | 1 | 1 | 1 | 1 |
| **Gender** |  |  |  |  |
| **Male** | 1 | 1 | 1 | 1 |
| **Female** | 1.04(0.99-1.08) | 1.10(1.04-1.16)** | 0.86(0.80-0.93)*** | 0.83(0.67-1.03) |
| **SES** |  |  |  |  |
| **Q1 – least deprived** | 1 | 1 | 1 | 1 |
| **Q2** | 1.07(0.98-1.16) | 1.13(1.02-1.25)* | 1.23(1.06-1.42)** | 1.43(0.96-2.14) |
| **Q3** | 1.09(1.00-1.19) | 1.17(1.05-1.31)** | 1.18(1.01-1.38)* | 1.23(0.8-1.9) |
| **Q4** | 1.14(1.05-1.24)** | 1.21(1.08-1.35)*** | 1.02(0.87-1.19) | 1.31(0.85-2.01) |
| **Q5** | 1.12(1.03-1.22)** | 1.14(1.03-1.27)* | 1.35(1.17-1.57)*** | 1.34(0.88-2.02) |
| **Q6** | 1.07(0.97-1.17) | 1.21(1.07-1.36)** | 1.11(0.94-1.31) | 1.37(0.87-2.15) |
| **Q7** | 1.05(0.95-1.15) | 1.16(1.04-1.31)* | 1.1(0.93-1.30) | 1.5(0.97-2.32) |
| **Q8** | 1.09(0.99-1.20) | 1.25(1.11-1.41)*** | 1.30(1.11-1.53)** | 1.2(0.76-1.89) |
| **Q9** | 1.00(0.90-1.09) | 1.31(1.16-1.48)*** | 1.33(1.13-1.57)*** | 1.12(0.69-1.81) |
| **Q10 – most deprived** | 1.10(0.99-1.22) | 1.22(1.08-1.38)** | 1(0.83-1.20) | 1.48(0.94-2.34) |
| **Smoking** |  |  |  |  |
| **Non-smoker** | 1 | 1 | 1 | 1 |
| **Ex-smoker** | 1.06(1.01-1.11)* | 1.11(1.05-1.18)*** | 1.01(0.93-1.09) | 1.39(1.11-1.74)** |
| **Current smoker** | 0.94(0.87-1.01) | 1.04(0.94-1.15) | 0.81(0.71-0.933)** | 2.23(1.6-3.1)*** |
| **BMI** | 1.01  (1.00-1.01)** | 1.02(1.01-1.02)*** | 1.03(1.02-1.03)*** | 1.01(0.99-1.03) |
| **Age** | 1.01  (1.01-1.01)** | 1.02(1.01-1.02)*** | 0.99(0.98-1)* | 1.09(1.06-1.11)*** |

Note:* p<0.1   ** p<0.05   *** p<0.01

**Table S15. Association between clusters of multimorbidity, health services utilisation and mortality, for the 85+ age group.**

|  | **1-year consultations** | **1-year new prescriptions** | **1-year hospital admissions** | **2-year mortality** |
| --- | --- | --- | --- | --- |
|  | **IRR (95% CI)** | **IRR (95% CI)** | **IRR (95% CI)** | **OR (95% CI)** |
| **Clusters** |  |  |  |  |
| **Lower morbidity better outcomes (Hypertension, chronic kidney disease, hearing loss)** | 1 | 1 | 1 | 1 |
| **Poor mental health (Painful condition, hypertension, hearing loss)** | 1.36(1.22-1.52)*** | 1.55(1.35-1.77)*** | 1.10(0.97-1.25) | 1.50(1.08-2.08)*** |
| **Established CVD (Hypertension, chronic hidney disease, coronary heart disease)** | 1.30(1.17-1.45)*** | 1.18(1.04-1.35)** | 1.05(0.93-1.19) | 1.99(1.40-2.55)*** |
| **Dementia-dominant (Painful condition, hypertension, constipation)** | 1.24(1.11-1.40)**** | 1.41(1.23-1.62)*** | 1.04(0.90-1.19) | 2.12(1.55-2.90)** |
| **Gender** |  |  |  |  |
| **Male** | 1 | 1 | 1 | 1 |
| **Female** | 1.02(0.93-1.10) | 1.04(0.94-1.15) | 0.89(0.81-0.98)* | 0.94(0.74-1.19) |
| **SES** |  |  |  |  |
| **Q1 – least deprived** | 1 | 1 | 1 | 1 |
| **Q2** | 1.13(0.99-1.29) | 1.36(1.15-1.6)*** | 1.01(0.85-1.19) | 1.38(0.92-2.08) |
| **Q3** | 1.11(0.96-1.28) | 1.42(1.19-1.70)*** | 1.08(0.9-1.29) | 1.40(0.9-2.16) |
| **Q4** | 1.12(0.96-1.31) | 1.41(1.17-1.7)*** | 1.04(0.86-1.25) | 1.70(1.09-2.66)* |
| **Q5** | 1.12(0.98-1.29) | 1.45(1.22-1.71)*** | 1.17(0.99-1.38) | 1.16(0.76-1.78) |
| **Q6** | 0.83(0.71-0.98)* | 1.11(0.9-1.36) | 0.9(0.73-1.12) | 1.29(0.79-2.08) |
| **Q7** | 1.06(0.90-1.24) | 1.66(1.37-1.97)*** | 1.03(0.85-1.24) | 1.24(0.77-1.99) |
| **Q8** | 1.17(0.99-1.39) | 1.61(1.32-1.97)*** | 1.16(0.95-1.41) | 1.44(0.88-2.37) |
| **Q9** | 0.90(0.75-1.08) | 1.48(1.18-1.85)** | 1.11(0.9-1.37) | 1.20(0.7-2.06) |
| **Q10 – most deprived** | 1.07(0.89-1.30) | 1.54(1.22-1.93)*** | 1.05(0.84-1.33) | 1.24(0.73-2.13) |
| **Smoking** |  |  |  |  |
| **Non-smoker** | 1 | 1 | 1 | 1 |
| **Ex-smoker** | 1.14(1.05-1.24)** | 1.13(1.02-1.25)* | 1(0.91-1.11) | 1.19(0.93-1.52) |
| **Current smoker** | 1.03(0.81-1.32) | 1.07(0.79-1.44) | 0.79(0.56-1.11) | 1.26(0.63-2.55) |
| **BMI** | 1.01(1.00-1.02)* | 1.01(1-1.02) | 1.01(1-1.02) | 0.99(0.96-1.01) |
| **Age** | 0.98(0.97-1.00)** | 1.01(0.99-1.02) | 0.99(0.98-1.01) | 1.06(1.03-1.09)*** |

Note:* p<0.1   ** p<0.05   *** p<0.01
